# Identification of yeast genes that confer resistance to chitosan oligosaccharide (COS) using chemogenomics

**DOI:** 10.1186/1471-2164-13-267

**Published:** 2012-06-22

**Authors:** Maria DLA Jaime, Luis Vicente Lopez-Llorca, Ana Conesa, Anna Y Lee, Michael Proctor, Lawrence E Heisler, Marinella Gebbia, Guri Giaever, J Timothy Westwood, Corey Nislow

**Affiliations:** 1 Department of Cell and Systems Biology, University of Toronto, Mississauga, Ontario, Canada; 2 Laboratory of Plant Pathology, Multidisciplinary Institute for Environmental Studies (MIES) Ramon Margalef, Department of Marine Sciences and Applied Biology, University of Alicante, Alicante, Spain; 3 Bioinformatics and Genomics Department, Prince Felipe Research Centre, Valencia, Spain; 4 Department of Molecular Genetics, University of Toronto, Toronto, Ontario, Canada; 5 Stanford Genome Technology Center, Palo Alto, California, USA; 6 Department of Pharmaceutical Sciences, University of Toronto, Toronto, Ontario, Canada; 7 Terrence Donnelly Centre for Cellular and Biomedical Research, University of Toronto, Toronto, Ontario, Canada; 8 Banting and Best Department of Medical Research, University of Toronto, Toronto, Ontario, Canada

**Keywords:** *Saccharomyces cerevisiae*, Chitosan oligosaccharide, Antifungal resistance, *ARL1*, Chemogenomics, Haploinsufficiency profiling (HIP), Homozygous profiling (HOP), Multi-copy suppression profiling (MSP), Transcriptional analysis, Stress response

## Abstract

**Background:**

Chitosan oligosaccharide (COS), a deacetylated derivative of chitin, is an abundant, and renewable natural polymer. COS has higher antimicrobial properties than chitosan and is presumed to act by disrupting/permeabilizing the cell membranes of bacteria, yeast and fungi. COS is relatively non-toxic to mammals. By identifying the molecular and genetic targets of COS, we hope to gain a better understanding of the antifungal mode of action of COS.

**Results:**

Three different chemogenomic fitness assays, haploinsufficiency (HIP), homozygous deletion (HOP), and multicopy suppression (MSP) profiling were combined with a transcriptomic analysis to gain insight in to the mode of action and mechanisms of resistance to chitosan oligosaccharides. The fitness assays identified 39 yeast deletion strains sensitive to COS and 21 suppressors of COS sensitivity. The genes identified are involved in processes such as RNA biology (transcription, translation and regulatory mechanisms), membrane functions (e.g. signalling, transport and targeting), membrane structural components, cell division, and proteasome processes. The transcriptomes of control wild type and 5 suppressor strains overexpressing *ARL1, BCK2, ERG24, MSG5,* or *RBA50*, were analyzed in the presence and absence of COS. Some of the up-regulated transcripts in the suppressor overexpressing strains exposed to COS included genes involved in transcription, cell cycle, stress response and the Ras signal transduction pathway. Down-regulated transcripts included those encoding protein folding components and respiratory chain proteins. The COS-induced transcriptional response is distinct from previously described environmental stress responses (i.e. thermal, salt, osmotic and oxidative stress) and pre-treatment with these well characterized environmental stressors provided little or any resistance to COS.

**Conclusions:**

Overexpression of the *ARL1* gene, a member of the Ras superfamily that regulates membrane trafficking, provides protection against COS-induced cell membrane permeability and damage. We found that the *ARL1* COS-resistant over-expression strain was as sensitive to Amphotericin B, Fluconazole and Terbinafine as the wild type cells and that when COS and Fluconazole are used in combination they act in a synergistic fashion. The gene targets of COS identified in this study indicate that COS’s mechanism of action is different from other commonly studied fungicides that target membranes, suggesting that COS may be an effective fungicide for drug-resistant fungal pathogens.

## Background

Chitin is an abundant natural polymer, second only in biomass to cellulose [1]. It is a common constituent of crustacean exoskeletons and arthropod cuticles [2] as well as the cell walls of most fungi [2,3]. There is an estimated 10 gigatons of chitin recycled in nature each year [4].

Chitosan is a polymer of N-glucosamine obtained by partial chitin N-deacetylation [1]. Acid hydrolysis or enzymatic cleavage of the glycosidic linkages of chitosan chains [5], yields shorter chains of <1-10 KDa chitosan oligosaccharides (COS). These polymers are less than 100 glucosamine monomers and are more water soluble and readily absorbed in vivo compared to chitosan [6].

Allan and Hadwiger [7] first showed the fungicidal effect of chitosan, and since then, several studies have examined chitosan sensitivity in different fungi [8-13]. Chitosan inhibits both hyphal growth and spore germination [14] and reduces toxin production by plant pathogenic fungi [15]. Recently, chitosan has been shown to increase conidiation in filamentous fungi at concentrations where hyphal growth was impaired [8]. Together these observations suggest that chitosan exerts its antifungal activity by multiple mechanisms.

We used COS in our study rather than chitosan because it is more soluble and biologically active. The biological activity of chitosan and COS is dependent on its molecular weight, degree of deacetylation and pH of the medium [16]. High degrees of deacetylation and an acidic pH (when most amino groups are in the free base form) yield the highest activity against susceptible fungi [17].

The antibiotic activity of chitosan and COS [16,18-20] is likely due to the permeabilization of bacterial plasma membranes [18,21]. Plasma membrane damage has also been suggested as an explanation of the fungicidal effects of chitosan in yeast and filamentous fungi [10,12,22,23]. Plasma membrane permeabilization by chitosan has also been detected (indirectly) in *Saccharomyces cerevisiae*, where deletion of genes encoding for proteins involved in maintaining plasma membrane integrity increased chitosan sensitivity [23]. Although it has been speculated that plasma membrane permeabilization by chitosan in bacteria and fungi is associated with the interaction between the positive amino groups of chitosan and the negative charges of phospholipids, no conclusive data are available [10,12,18].

To gain a better understanding of the mode of action of small (< 6 kDa) chitosan oligosaccharides (COS-5.44), and identify putative resistance mechanisms, we performed three chemogenomic screens [24,25]. These assays examined the effect of gene dosage to uncover *bona fide* targets of COS in yeast. Two chemogenomics assays interrogate the yeast deletion collection (~ 6000 deletion strains) in a single culture in parallel. Each deletion strain in this collection contains a unique 20-base-pair DNA tag used to quantify the fitness of individual strains using an oligonucleotide array [24,26]. Both essential heterozygous and non-essential homozygous diploids were assayed. The haploinsufficiency profiling (HIP) assay identifies genes that show increased drug sensitivity in heterozygous deletion strains. The heterozygous deletion strain that is most sensitive to a given compound often indentifies its target [24,26]. The homozygous profiling (HOP) assay identifies genes that buffer the drug target pathway and are required for resistance to the compound [24,26]. A multicopy suppression profiling (MSP) screen, where genes are overexpressed to identify those that confer resistance to the compound of interest was also performed [25]. The genes identified from the chemogenomic assays were individually validated to confirm their involvement in COS sensitivity or resistance. For five of the strains that conferred resistance when overexpressed, we performed a transcriptional analysis. The *ARL1 (ADP-ribosylation factor-like 1)* gene was identified in the HIP-HOP and MSP chemogenomics assays as being involved in yeast resistance to COS-5.44. This gene encodes a soluble GTPase that is a member of the Ras superfamily and has been shown to be involved in membrane traffic regulation [27,28]. *ARL1* overexpression strains showed decreased COS-induced permeability compared to wild type cells. Because COS is a cell stress causing agent, we compared the transcriptional response induced by COS to those of other environmental stressors and ascertained whether an environmental stress pretreatment could provide resistance to COS. Finally, we evaluated whether a COS-resistant *ARL1* overexpressing strain is also resistant to other antifungal compounds.

## Results

### Sensitivity to chitosan and COS-5.44 treatment in the yeast deletion pools

**Figure 1:**
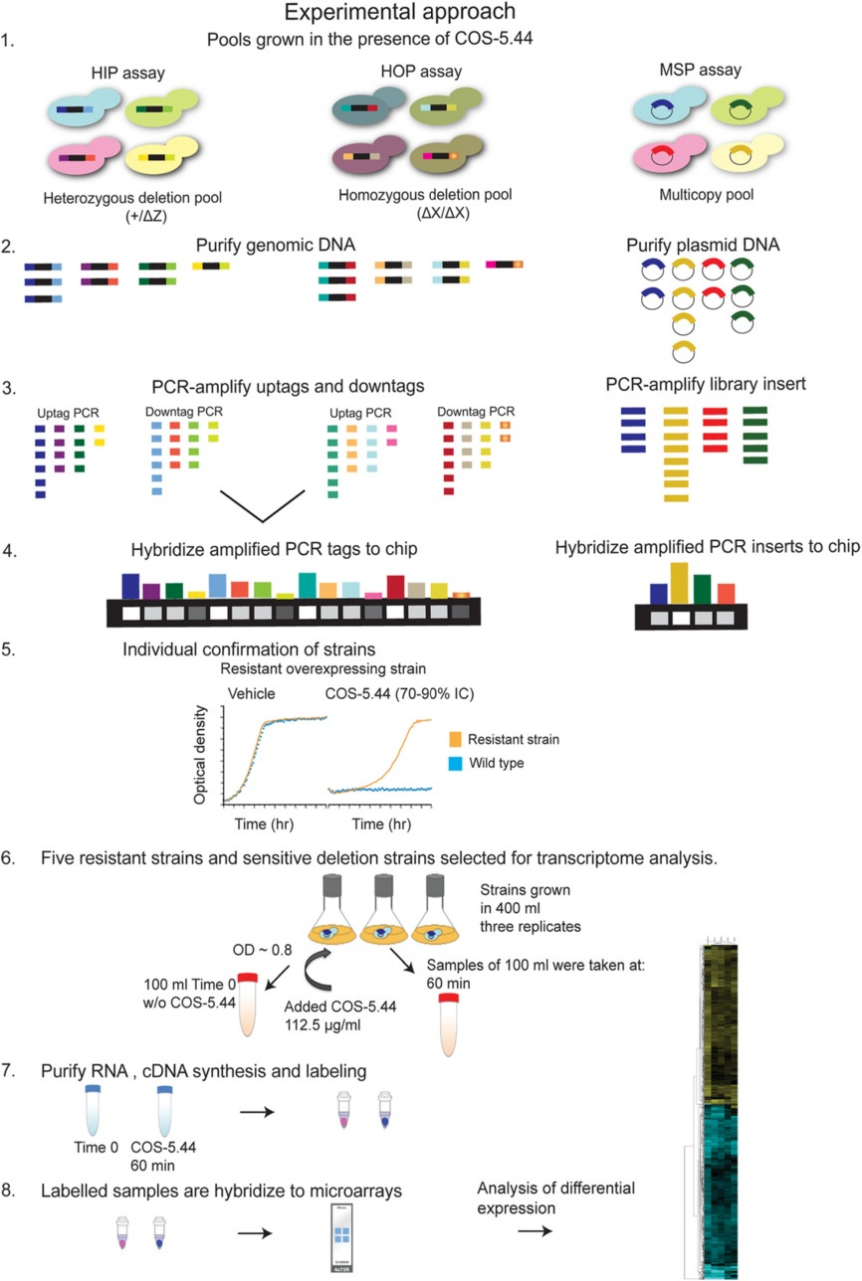
**Experimental approach used to identify and characterize genes that confer resistance to chitosan oligosaccharide (COS). **Three chemogenomic assays were used: Haploinsufficiency profiling (HIP), homozygous profiling (HOP) and multicopy suppression profile (MSP). (1) Heterozygous, homozygous deletion pools and multicopy suppression pool were grown competitively in the presence of COS-5.44. If a gene is required to grow in the presence of COS, the corresponding deletion strain will grow more slowly and therefore will be underrepresented. Cells overexpressing a gene that suppresses sensitivity will growth faster and will be overrepresented in the MSP pool. (2) Genomic DNA was isolated from cells prior to and after the HIP-HOP assays, and plasmid purification from the COS treated MSP pool was carried out. (3) Barcodes were PCR amplified for HIP-HOP assays as well as the plasmid inserts of MSP. (4) PCR products (barcodes and plasmid inserts) were hybridized to a TAG4 array. Intensity of treatment samples is compared with intensity of a control sample to determine relative abundance (~ fitness). (5) Sensitive deletion strains and constructed overexpressing strains were individually confirmed. (6) Five resistant overexpressing strains that were also sensitive as deletion strains were selected for transcriptome analysis. Overexpressing strains and vector control were grown in the presence of COS (112.5 μg/ml) and cells were harvested before COS treatment and after 60 min of COS treatment. (7) RNA was isolated from harvested cells, cDNA synthesized and labelled with fluorescent dyes. (8) Labelled samples were hybridized to expression microarrays. Transcriptional changes were indentified by differential expression analysis. Figure modified from Ericson et al. (2010).

**Figure 2:**
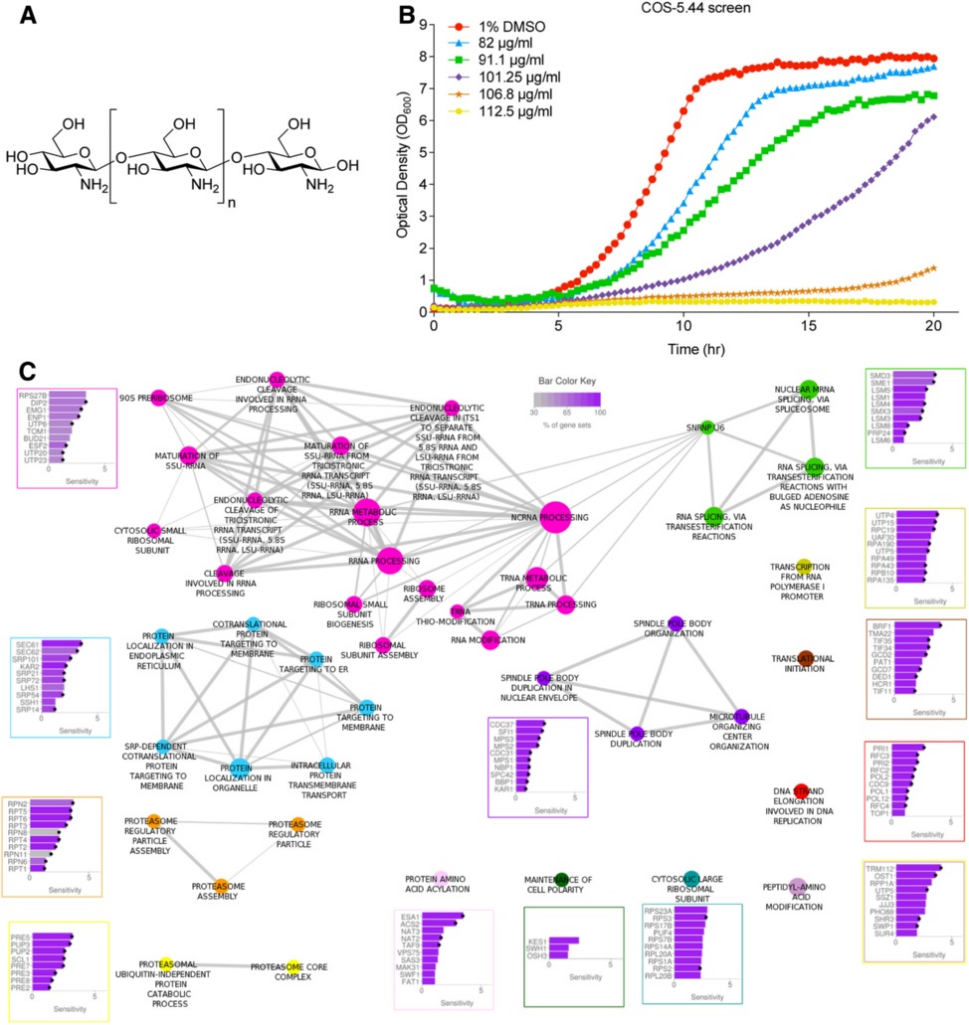
**Chitosan oligosaccharide molecule, screen and biological process associated with sensitive deletions strains. A**) Chitosan molecular structure. **B**) Chitosan oligosaccharide screen in wild type yeast (BY4743) at 5 concentrations (91.1 – 112.5 μg/ml) in 0.5X YPD pH 5. Optical density readings were taken every 15 min over 20 hrs using a Tecan Genios reader (see Methods). **C**) Biological processes associated with chitosan oligosaccharide (COS) sensitive deletion strains. A node represents a biological process significantly enriched in the COS-5.44 HIP-HOP assay (FDR ≤ 0.1, see Methods). The node size correlates to the number of genes annotated to that functional category. The width of the edge correlates to the degree of gene overlap between the 2 connected categories. If the overlap coefficient is less than 0.5, edges are not shown (see Methods). Cluster membership is shown by node color, where clustering is based in degree of overlap among categories. Bar plots show the sensitivity scores of the genes that contributed to the functional enrichment of the cluster, border color surrounding the plot correlated with the nodes in the cluster. The top 10 genes in each cluster category are shown in each plot. Heterozygous strains are marked with a black dot.

A scheme summarizing the experiments and analyses performed in this study is shown in Figure 1. We first compared yeast sensitivity to deacetylated chitosan (approximately 70 kDa, 80% deacetylated) and to chitosan oligosaccharide (COS) (5.44 kDa, 97% deacetylated). COS (Additional file 1: Figure S1A) had considerably higher antifungal activity than chitosan at equivalent concentrations (Additional file 1: Figure S1B). Because of this, it is easier to more consistently manufacture this form, we decided to use COS as the antifungal agent. The heterozygous and homozygous yeast deletion collections were grown in YPD with 91.1 μg/ml COS-5.44, a concentration that inhibited yeast wild type growth by 10 – 20% compared with the control (Figure 2B). In the HIP-HOP assays, 39 yeast deletion strains sensitive to COS-5.44 were indentified (22 homozygous and 17 heterozygous; log2 ratio ≥ 3.5, see Methods; Table 1, Additional file 2: Table S1).

**Table 1:** Top 39 characterized sensitive deletion strains found in the HIP-HOP assays above 3.5 log 2 ratio

**probeid**	**COS-5.44 (91.1 μg/ml) (log2ratio)**	**Gene**	**SGD_description**	**Fitness score (avg)**^**2**^	**Deletion pool source**^**3**^
YBR 164C	4.686	ARL1	Soluble GTPase of the RAS superfamily, with a role in regulation of membrane traffic mainly regulates pottassium influx; similar to ADP-ribosylation factor.	0.756	**HOM**
YBR257W	3.534	POP4	subunit of both RNase MRP, which cleaves pre-rRNA, and nuclear RNase P, which cleaves tRNA precursors to generate mature 5′ends; binds to the RPR1 RNA subunit in RNase P.	0.9963	**HET**
YDL060W	3.769	TSR1	Protein required for processing of 20S pre-rRNA in the cytoplasm associates with pre-40S ribosomal particles.	0.8887	**HET**
YDL213C	5.101	NOP6	rRNA-binding protein required for 40S ribosomal subunit biogenesis; contains an RNA recognition motif (RRM).	0.8854	**HOM**
YDR300C	4.371	PRO1	Gamma-glutamyl kinase, catalyzes the first step in proline biosynthesis.	0.8521	**HOM**
YDR320C	3.571	SWA2	Auxilin-like protein involved in vesicular transfort; clathrin-binding protein required for uncoating of clathrin-coated vesicles	0.9621	**HOM**
YDR324C	3.636	UTP4	Subunit of U3 involved in production of 18S rRNA, assembly of small ribosomal subunit and transcription of 35S rRNA transcript, member of t-Utp subcomplex.	0.761	**HET**
YDR389W	4.281	SAC7	GTPase activating protein (GAP) for Rho1p, involved in signaling to the actin cytoskeleton, null mutations suppress tor2 mutations and temperature sensitive mutations in actin; potential Cdc28p substrate.	0.8768	**HOM**
YDR455C	3.577	YDR455C	Partially overlaps the verified gene YDR456W.	0.9312	**HOM**
YGL045W	4.229	RIM8	Protein involved in proteolytic activation of Rim 101p in response to alkaline pH; interacts with ESCRT-1 subunits Stp22p and Vps28p; member of the arrestin-related trafficking adaptor family.	0.896	**HOM**
YGR056W	3.72	RSG1	Component of the RSC chromatin remodeling complex; required for expression of mid-late sporulation-specific genes.	0.8656	**HOM**
YGR122W	3.719	YGR122W	Probable ortholog of A. nidulans PalC, which is involved in pH regulation and binds to the ESCRT-III complex; null mutant does not properly process Rim 101p and has decreased resistance to rapamycin.	0.9913	**HOM**
YGR246C	3.887	BRF1	TFFIIIB B-related factor, one of three subunits of RNA polymerase III transcription initiation factor TFFIIIB, binds TFIIIC and TBP and recruits RNA pol III to promoters, amino-terminal half is homologous to TFIIB.	0.9782	**HET**
YHL009C	3.625	YAP3	Basic leucine zipper (bZIP) transcription factor.	0.8854	
YIL019C	3.59	FAF1	Protein required to pre-rRNA processing and 40S ribosomal subunit assembly.	0.9603	**HET**
YIL022W	3.931	TIM44	Essential component of the Translocase of the inner Metochondrial membrane; tethers the import motor and regulatory factors (PAM complex) to the translocation channel (Tim23p-Tim17p core complex).	0.8167	**HET**
YIL048W	3.83	NEO1	Putative aminophospolipid translocase (flippase) involved in endocytosis and vacuolar biogenesis.	1.0734	**HET**
YIL075C	3.653	RPN2	Subunit of the 26Sproteasome, substrate of the N-acetyltransferase Nat1p.	0.9832	**HET**
YIL154C	4.315	IMP2	Transcriptional activator involved in maintenance of ion homeostasis and protection against DNA damage caused by bleomycin and other oxidants.	0.2516	**HOM**
YJL002C	3.515	OST1	Alpha subunit of the oligosaccharyltransferase complex of the ER lumen, which catalyzes asparagine-linked glycosylation of newly synthesized proteins.	1.0555	**HET**
YGL188C	3.656	BUD19	ORF overlaps the verified gene RPL39 by 88%f; diploid mutants displays a weak budding pattern phenotype in a systematic assay.	1.0087	**HOM**
YJR102C	3.755	VPS25	Component of the ESCRT-II complex, which is involved in ubiquitin -dependent sorting of proteins into the endosome.	1.0011	**HOM**
YKL114C	4.271	APN1	Apurinic/apyrimidinic endonuclease, 3′-repair diesterase involved in repair of DNA damage by oxidation and alkylating agents; also functions as a 3′-5′ exonuclease to repair 7,8-dihydro-8-oxodeoxyguanosine.	0.8878	**HOM**
YKR024C	3.506	DPB7	Putative ATP-dependent RNA helicase of the dead -box family involved in ribosomal biogenesis.	0.9795	**HOM**
YKR025W	4.622	SNF7	One of four subunits of the endosomal sorting complex required for transport III (ESCRT-III); involved in the sorting of transmembrane proteins into the multivesicular body (MVB) pathway.	0.8599	**HOM**
YLR100W	3.929	ERG27	3-keto sterol reductase, catalyzes of the last three steps required to removed two C-4 methyl groups from an intermediate in engosterol biosynthesis; mutants are sterol auxotrophs.	0.8809	**HET**
YLR147C	3.525	SMD3	Core Sm protein Sm D3; part of heteroheptameric complex that is part of the spliceosomal U1, U2, U4, and U5 snRNPs; homolog of human Sm D3.	1.0582	**HET**
YLR223C	3.633	IFM1	Coactivator that regulates transcription of ribosomal protein (RP) genes; recruited to RP gene promoters during optimal growth conditions *via* Fhl1p; subunit of CURI, a complex that coordinates RP production and pre-rRNA processing.	0.9691	**HET**
YLR378C	3.539	SEC61	Essential subunit of Sec61 complex (Sec61p, Sbh1p, and Sss1p); with Sec63 complex forms a channel for SRP-dependent protein import and retrograde transport of misfolded proteins out of the ER.	0.8717	**HET**
YNL025C	3.581	SSN8	Cyclin-like component of the RNA polymerase II holoenzyme, involved in phosphorylation of the RNA polymerase II C-terminal domain, in glucose repression and telomere maintenance.	0.823	**HOM**
YNL287W	3.66	SEC21	Gamma subunit of coatomer, a heptameric protein complex that together with Arf1p forms the COPI coat; involved in ER to Golgi transport of selective cargo.	0.8959	**HET**
YNR046W	4.047	TRM112	Subunit of tRNA methyltransferase (MTase) complexes in combination with Trm9p and Trm11p; subunit of complex with Mtq2p that methylates Sup45p (eRF1) in the temary complex eRF1-eRF3-GTP.	0.9906	**HET**
YOR014W	3.844	RTS1	B-type regulatory subunit of protein phospatase 2A (PP2A); homolog of the mammalian B’subunit of PP2A.	0.9832	**HOM**
YOR030W	3.669	DFG16	Probable multiple transmembrane protein, involved in diploid invasive and pseudohyphal growth upon nitrogen starvation; required for accumulation of processed Rim101p.	1.0307	**HOM**
YOR080W	4	DIA2	Origin-binding F-box protein that forms an SCF ubiquitin ligase complex with Skp1p and Cdc53p; plays a role in DNA replication, involved in invasive and pseudohyphal growth.	1.1312	**HOM**
YOR117W	3.506	RPT5	One of the six ATPases of the 19S regulatory particle (26S proteasome) involved in the degradation of ubiquintinated substrates; recruited to the GAL1-10 promoter region upon induction of transcription; similar to human TBP1.	0.8955	**HET**
YOR244W	3.5	ESA1	Catalytic subunit of the histone acetyltransferase complex (NuA4) that acetylates four conserved internal lysines of histone H4N-terminal tail; required for cell cycle progression and transcriptional silencing at the rDNA locus.	0.8716	**HET**
YPL002C	3.679	SNF8	Component of the ESCRT-II complex, which is involved in ubiquitin-dependent sorting of proteins into the endosome; appears to be functionally related to SNF7; involved in glucose derepression.	0.8925	**HOM**
YPR139C	3.643	VPS66	Cytoplasmic protcin of unknown function involved in vacuolar protcin sorting.	0.8768	**HOM**

^1^ COS-5.44 log2 ratio is calculated from tag4 array data for each tag of each deletion strain. COS-5.44 log2 ratio = log2 (μ_c_ - *bg*)/(μ_t_ - *bg*), where μ_c_ is the mean intensity of the control sample, μ_t_ is the mean intensity of the treated samples, and *bg* is the mean intensity of the unassigned probes. This score is proportional to the log2 ratio of cells present in the control sample *versus* the treatment sample (i.e. deletion strains exposed to 91.1 μg/ml of COS) [24].

^2^ Fitness score *(W*) is estimated from the average growth curves (as measured by optical density) of a given strain in the presence and absence of COS after 20 h. The fitness scores from three individual colonies of each deletion strain grown in triplicate (i.e. 9 cultures for each strain) were used to calculate the average. This score takes into account the growth of the wild type and deletion strain in control conditions as well as treatment conditions. *W*_*c*_ = wt_c_/st_c_; Wt = wt_t_/st_t_, where *W*_*c*_ is the fitness score under control (no drug) conditions, *W*_*t*_*is the* fitness score after treatment, wt is doubling time of wild type strain, st is doubling time of the deletion strain, c is control conditions, t is treatment (COS) conditions. Normalized fitness (*W*) = avg W_t_/avg W_c_[24]. A fitness score less than 1 indicates that the strain is sensitive [24]. A minimum difference of 0.1 (10%) was used as a cut-off to consider a strain sensitive.

^3^ Deletion pool source identifies which pool of deletion strains the deleted strain came from. HET - heterozygous essential deletions strains, HOM - homozygous deletion strains.

A previous global fitness analysis, similar to the HIP-HOP assay we performed, identified 101 chitosan sensitive homozygous and three heterozygous deletion strains, [23]. Approximately 10% of the homozygous deletion strains found in their study were among the genes identified in our screen (*ARL1*, *IMP*2’, *APN1*, *RSC1*, *SNF8*, *DFG16*, *VPS66*, *YAP3*, *SSN8*), but none of the three heterozygous deletions strains corresponded to those identified in our screen.

Biological processes (defined by Gene Ontological terms) associated with the COS-5.44 sensitive deletion strains were determined using gene set enrichment analysis (GSEA) [29]. The enriched processes included RNA biology (transcription, translation and regulatory mechanisms), membrane functions (e.g. transport and targeting), membrane structural components (e.g. proteins), cell division (spindle body and microtubules) and proteasome processes (structural and regulatory functions; Figure 2C, FDR ≤ 0.1, see Methods). Similar GO terms showed altered fitness in the presence of chitosan in a similar fitness analysis [23].

To confirm whether the 39 deletion strains were sensitive to COS on their own, we tested each strain individually and found that 21 (~50%) were inhibited by COS (Table 1).

### Resistance to COS-5.44 treatment in the multicopy suppression profiling pool

MSP assays involve the transformation of yeast with a multicopy suppression plasmid library containing random fragments of approximately 7 kbp pieces of DNA. The MSP assay was performed with 101.25 – 250.0 μg/ml COS-5.44 and it was determined that 112.5 μg/ml COS-5.44 inhibited wild type yeast growth by at least 70% (Figure 2B). Yeast containing suppression plasmids that provide resistance to 112.5 μg/ml COS-5.44 will expand more quickly than the rest of the yeast of the population. Genomic DNAs from a resistant population and a transformed but untreated population are extracted and hybridized to ORF microarrays to identify genes present in multicopies in the resistant population.

Two independent replicates of the MSP screen identified a total of 68 genes as putative suppressors of COS-5.44 sensitivity (Additional file 1: Figure S2). The random genomic DNA fragments in the multicopy suppression plasmid library contain on average 2 – 3 yeast genes per fragment [25]. Only one gene in each fragment is likely responsible for resistance; therefore we expected only 33-50% of these genes to be confirmed as suppressors of COS-5.44 (see below and Additional file 2: Table S2). There were 42 and 32 suppressing genes in replicates 1 and 2 respectively with only 6 genes in common (Additional file 1: Figure S2). The 6 genes were: *PKR1*, a V-ATPase assembly factor in the endoplasmic reticulum (ER) which functions with other V-ATPase assembly factors to efficiently assemble the V-ATPase membrane sector; *COX5A*, subunit Va of cytochrome c oxidase, involved in mitochondrial electron transport; *FLO1*, a lectin-like cell wall protein involved in flocculation that binds to mannose on the surface of other cells; *VAC7*, a vacuolar membrane protein involved in inheritance and vacuole morphology; *RPL20A*, a large ribosomal subunit protein; and *MSG5*, a dual specificity phosphatase protein required for maintenance of low level signaling through the cell integrity pathway which regulates and is regulated by Slt2p. Three of these genes, *PKR1*, *VAC7* and *MSG5*, were also found to be sensitive to COS when tested as single heterozygous deletion strains. Among the suppressing genes found in replicate 2, *ARL1,* a gene encoding a GTPase protein, was identified. This gene was found to be sensitive to COS-5.44 as a deletion strain in the HIP-HOP assay (Table 1).

### Confirmation of COS suppressing strains

**Figure 3:**
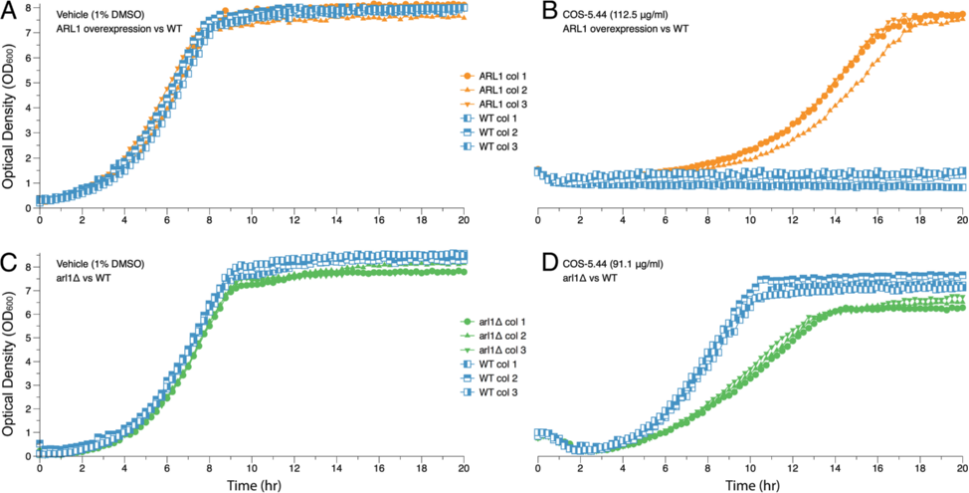
**Confirmation of COS-5.44 resistance in the *Arl1 *overexpressing strain and sensitivity of the corresponding heterozygous deletion strain.** The overexpression of *ARL1* does confer resistance to COS-5.44 (112.5 μg/ml) compared with the wild type (vector control) that is not able to grow at this concentration. The heterozygous deletions strain (*arl1*Δ) shows sensitivity to COS-5.44 (91.1 μg/ml). **A**) Growth curves of *ARL1* and wild type (vector control) growth in the vehicle. **B**) Growth curves of *Arl1* overexpressing and wild type strains growth with COS-5.44 (112.5 μg/ml). **C**) Growth curves of the heterozygous deletion (*arl1*Δ) and wild type strain grown with the vehicle. **D**) Growth curves of heterozygous deletion (*arl1*Δ) and wild type strains grown with COS-5.44 (91.1 μg/ml). Optical density readings were taken every 15 min over 20 hrs using a Tecan Genios reader. Tecan ODs were converted to conventional 1 mm path length cuvette ODs using a calibration function provided by Ericson et al. 2010, [24]. Similar behaviour was observed for the rest of the selected overexpressing strains (*Bck2*, *Erg24*, *Msg5* and *Rba50*) deduced to be resistant to chitosan oligosaccharide COS-5.44 from the MSP screen. The heterozygous deletion strains from these genes (*bck2*Δ*, erg24*Δ*, msg5*Δ *and rba50*Δ) were also individually tested for sensitivity to COS-5.44 (91.1 μg/ml). Three colonies of each strain were grown in triplicate and compared with the wild type in the presence of the COS-5.44 and the vehicle (1% DMSO).

After the identification of putative resistant strains we tested each strain individually. From the 68 candidate suppressor genes, 57 individual overexpressing/suppressing strains each containing a single putative resistance gene were obtained from a collection of ORFs whose expression is driven by native promoters [30] see Methods. Twenty-one (~31%) of the putative suppressor genes conferred resistance to COS-5.44 when tested in this manner (Table 2). COS-5.44 resistant overexpressing strains were able to grow in 112.5 μg/ml COS-5.44 while the wild type (transformed with an empty vector) was unable to grow (Figure 3B).

**Table 2:** Twenty-one yeast overexpression strains confirmed as suppresors of sensitivity to COS-5.44

**ORF**	**Gene**	**Resistance confirmed**	**Fitness score of resistance (avg)**^**3**^	**Sensitivity confirmed (HET deletion strain)**	**Fitness score of sensitivity (avg)**^**4**^	**Description from SGD**
YBR164C	ARL1^1^	yes	1.870	yes	0.756	Soluble GTPase of the Ras superfamily, with a role in regulation of membrane traffic mainly regulates potassium influx; similar to ADP-ribosylation factor.
YBR166C	TYR1	yes	1.381	no	1.035	Prephenate dehydrogenase involved in tyrosine biosynthesis, expression is dependent on phenylalanine levels.
YDL029W	ARP2	yes	1.609	no	1.023	Essential component of the Arp2/3 complex involved in endocytosis and membrane growth and polarity. A conserved actin nucleation center required for the motility and integrity of actin patches.
YDR171W	HSP42	yes	1.372	no	1.135	Small heat shock protein (sHSP) with chaperone activity involved in cytoskeleton reorganization after heat shock; forms barrel-shaped oligomers that suppress unfolded protein aggregation.
YDR524C	AGE1	yes	1.783	no	1.106	ADP-ribosylation factor (ARF) GTPase activating protein (GAP) effector, involved in the secretory and endocytic pathways.
YDR527W	RBA50^1^	yes	1.407	yes	0.899	Protein involved in transcription; interacts with RNA polymerase II subunits Rpb2p, Rpb3, and Rpb11p; has similarity to human RPAP1.
YER048C	CAJ1	yes	1.845	no	0.995	Nuclear type II J heat shock protein of the of the E. coli dnaJ family, binds, to non-native substrates for presentation to Ssa3p, may function during protein translocation, assembly and disassembly.
YER167W	BCK2^1^	yes	1.365	yes	0.890	Protein rich in serine and threonine residues involved in protein kinase C signaling pathway, which controls cell integrity; overproduction suppresses pkc 1 mutations
YJL046W	AIM22	yes	1.258	no	0.908	Putative lipoate-protein ligase, required along with Lip2 and Lip5 for lipoylation of Lat1p and Kgd2p.
YKL208W	CBT1	yes	1.265	no	0.930	a role in 3′end processing of the COB pre-mRNA; displays genetic interaction with cell cycle-regulated kinase.
YLR193C	UPS1	yes	1.201	no	0.953	Mitochondrial intermembrane space protein that regulates mitochondrial cardiolipin levels, null has defects in Mgm1p processing, integrity of mitochondrial inner membrane complexes; ortholog of human PRELI.
YLR285W	NNT1	yes	1.448	no	1.184	Putative nicotinamide N-methyltransferase, has a role in rDNA silencing and in the lifespan determination.
YML124C	TUB3	yes	1.763	no	1.227	Alpha-tubulin; associates with beta-tubulin (Tub2p) to form tubulin dimer, which polymerizes to form microtubules; expressed at lower level than Tub1p.
YMR123W	PKR1^2^	yes	1.350	yes	0.866	V-ATPase assembly factor, functions with other V-ATPase assembly factors in the ER to efficiently assemble the VATPase membrane sector (VO).
YNL053W	MSG5^1^	yes	1.454	yes	0.874	Dual-specificity protein phosphatase; required for maintenance of a low level of signaling through the cell integrity pathway, adaptive response to pheromone; regulates and is regulated by Slt2p; dephosphorylates Fus3p.
YNL054W	VAC7^2^	yes	1.387	yes	0.873	Integral vacuolar membrane protein involved in vacuole inheritance and morphology; activities Fab1p kinase activity under basal conditions anfd also after hyperosmotic shock.
YNL218W	MSG1^2^	yes	1.214	yes	0.899	Protein with DNA-dependent ATPase and ssDNA annealing activities involved in maintenance of genome; interacts functionally with DNA polymerase delta; homolog of human WHIP.
YNL280C	ERG24^1^	yes	1.275	yes	0.893	C-14 sterol reductase, acts in ergosterol biosynthesis; mutants accumulate the abnormal sterol ignosterol (ergosta-8, 14 dienol).
YNR057C	BIO4^2^	yes	1.246	yes	0.885	Dethiobiotin synthetase, catalyzes the third step in the biotin biosynthesis pathway; BIO4 is in a cluster of 3genes (BIO3, BIO4, and BIO5) that mediate biotin synthesis.
YPL053C	KTR6^2^	yes	1.616	yes	0.861	Probable mannosylphosphate transferase involved in the synthesis of core oligosaccharides in protein glycosylation pathway; member of the KRE2/MNT1 mannosyltransferase family.
YPL106C	SSE1^2^	yes	1.253	yes	0.896	ATPase that is a component of the heat shock protein Hsp90 chaperone complex; binds unfolded proteins; member of the heat shock protein 70 (HSP70) family; localized to the cytoplasm.

^1^ Genes selected for transcriptomic analysis.

^2^ Overexpressing strains confirmed as suppressors when overexpressed and sensitive when tested as deletion strains.

^3–4^ Fitness score *(W*) is estimated from the average growth curves (as measured by optical density) of a given strain in the presence and absence of COS after 20 h. Data shown corresponds to deletion strains exposed to 91.1 μg/ml and overexpressing strains to 112.5 μg/ml of chitosan oligosaccharide. The fitness scores from three individual colonies of each overexpressing or deletion strain grown in triplicate (i.e. 9 cultures for each strain) were used to calculate the average. This score takes into account the growth of the wild type and overexpressing or deletion strain in control conditions as well as treatment conditions. *W*_*c*_ = wt_c_/st_c_; Wt = wt_t_/st_t_, where *W*_*c*_ is the fitness score under control (no drug) conditions, *W*_*t*_*is the* fitness score after treatment, wt is doubling time of wild type strain, st is doubling time of the overexpressing or deletion strain, c is control conditions, t is treatment (COS) conditions. Normalized fitness (*W*) = avg W_t_/avg W_c_[24]. A fitness score less than 1 indicates that the strain is sensitive, a fitness score above 1 indicates that the strain is resistant [24]. Means that the strain has a treatment induced growth defect, a minimum difference of 0.1 (10%) was used as a cut-off to consider a strain sensitive.

Among the confirmed genes that confer resistance to COS-5.44 were two essential genes: *RBA50* (YDR527W), a gene whose protein product is involved in transcription, and *ARP2* (YDL029W), a gene encoding actin related protein 2, a subunit of the Arp2/3 complex which is required for the motility and integrity of cortical actin patches. Among the remaining 19 non-essential genes, a diversity of functions were found, such as a sterol reductase, a heat shock protein, ADP-ribosylation factors, soluble GTPases, alpha tubulin, and a ubiquitin conjugation enzyme. Putative suppressors of COS antifungal action in yeast were all tested as heterozygous deletion strains and from the 21 resistant overexpressing strains, 11 were found to be sensitive when tested as heterozygous deletion strains (Table 2). Five genes that provided COS resistance when overexpressed or sensitivity as a heterozygous or homozygous deletion strain were selected for further study primarily based on the known or putative functions of the genes. More specifically we selected genes with roles in signalling pathways, cell or membrane integrity, or transcription regulation.

We selected the following 5 overexpressing strains: *ARL1*, which encodes a GTPase involved in membrane trafficking, was selected because it was found in the HIP-HOP assay as a sensitive homozygous deletion strain and in the MSP assay as a multicopy suppressor. The other overexpressing strains we selected were: *BCK2* and *MSG5*, which are both involved in cell integrity pathways; *ERG24,* a gene involved in ergosterol synthesis; and *RBA50* (mentioned above). *BCK2* is a Ser-Thr rich protein with protein kinase C activity that acts in signal transduction. Overexpression of *BCK2* can rescue defects in a *cwh43Δ* mutant that displays several cell wall defects [31]. *BCK2* overexpression can also suppress the cell lysis defects seen when the kinases *Mpk1* and *Pck1* are deleted [32]. *MSG5* encodes a protein phosphatase involved in cell cycle control through the dephosphorylation of MAPK and is required for restricting signaling by the cell integrity pathway in yeast [33]. The inhibition of MAPK signaling leads to inhibition of cell differentiation and cell division [34]. The functions of *ARL1* and *ERG24* and their potential roles in chitosan resistance are described in more detail in the Discussion.

The fitness of all overexpressing strains compared to an empty vector control and the corresponding heterozygous deletion strains were assessed. In the presence of COS-5.44, the overexpressing strains always grew better than the vector control (Figure 3B, showing results of *ARL1*) and the corresponding deletion strain demonstrated reduced fitness (Figure 3D). In the absence of the COS-5.44, no growth differences were observed among each overexpressing strain or deletion strains (tested in triplicate) compared with the vector control, indicating the absence of inherent fitness defects (Figure 3A and 3C).

### Transcriptional response to COS

To identify the transcriptional changes caused by exposure to COS-5.44, wild type (vector control) cells, were exposed to COS for 1 h, total RNA extracted, and a microarray analysis was performed. A total of 335 genes were differentially expressed in response to COS-5.44 (P-value < 0.05 and log2 fold change > 1 or < −1).

A GSEA was performed for the entire transcription data set and among the up-regulated transcripts, we identified cellular respiration, ATP production, protein complex biogenesis, and mitochondria translation and organization as enriched biological processes (*P* < 0.005). For the down-regulated transcripts, the enriched terms included glycosylation, transmembrane transport, and sterol and lipid biosynthesis. For the overexpressing strains that provided resistance to COS (see below), the majority of these biological processes had the opposite characteristic (i.e. enriched biological processes among up-regulated transcripts in the wild type were enriched among down-regulated transcripts in the overexpression strains). A comparison of the transcriptional profiles of the COS-5.44 treated wild type cells in this study with the chitosan treated 60 min profile from another similar study, [22] was performed (Additional file 1: Figure S3, Additional file 2: Table S13). As might be expected, there was considerable overlap among enriched biological processes between the two studies (Additional file 1: Figure S3, Additional file 2: Table S13), and included such terms as cell wall organization, ATP production, and oxidative phosphorylation as being enriched among up-regulated transcripts. Ribosome biogenesis and polyphosphate metabolic processes were enriched among down-regulated transcripts. There were also several biological processes that showed up as having opposite enrichments in the two studies. For example, sterol/ergosterol/lipid biosynthesis was observed as a down-regulated process in our study while these were up-regulated in Zakrzewska and co-workers study. While it is not clear why some processes show opposite enrichments in the two studies, we do know that different types and amounts of anti-fungal agents were used, Zakrzewska et al. exposed the yeast cells to fragmented chitosan at an IC_10 - 15_ and in this study, cells were exposed to COS-5.44 at an IC_70 – 80_.

### Transcriptome analysis of the COS-5.44 resistant strains

To gain a further understanding on the mode of action and mechanisms of resistance to COS, we performed a transcriptome analysis of the 5 overexpressing strains known to increase resistance to COS-5.44 (data available in GEO under GSE32888). Each overexpressing strain and the wild type (vector control) were treated with COS-5.44 for 1 h or mock treated with vehicle alone and RNA was isolated from each sample. The RNA was converted to labeled cDNA and hybridized to NimbleGen expression microarrays (see Methods). We performed two sets of analyses. The first analysis was designed to find genes differentially expressed genes in each overexpression strain compared to wild type in the absence of COS in order to see if overexpression of the gene in question changed the transcriptional profile (and potentially the physiology or structure of the cell). The second analysis was designed to find differentially expressed genes in each overexpression strain compared to wild type in the presence of COS to see if the overexpression strains responded differently to COS than wild type.

In the first analysis, we identified 184 genes with differential expression in at least one of the 5 overexpressing strains compared with the wild type (vector control) without COS-5.44 treatment (Additional file 1: Figure S4, Additional file 2: Table S3; P-value ≤ 0.05 and log2 fold change ≥ 1 or ≤ −1). Each of the 5 overexpressing strains showed increased expression of the gene contained in the transformation vector (Additional file 1: Figure S4 A-D). There was a subset of 13 highly up-regulated genes in the *ARL1* and *RBA50* strains (Additional file 1: Figure S4 A) including genes encoding heat shock proteins, acid phosphatases, inorganic phosphatases, and transmembrane transporters. Several genes involved in oxidative phosphorylation, amino acid and carbohydrate metabolic processes and biosynthesis, and mitochondrial ATP synthesis and electron transport were up-regulated (Additional file 1: Figure S4 D).

**Figure 4:**
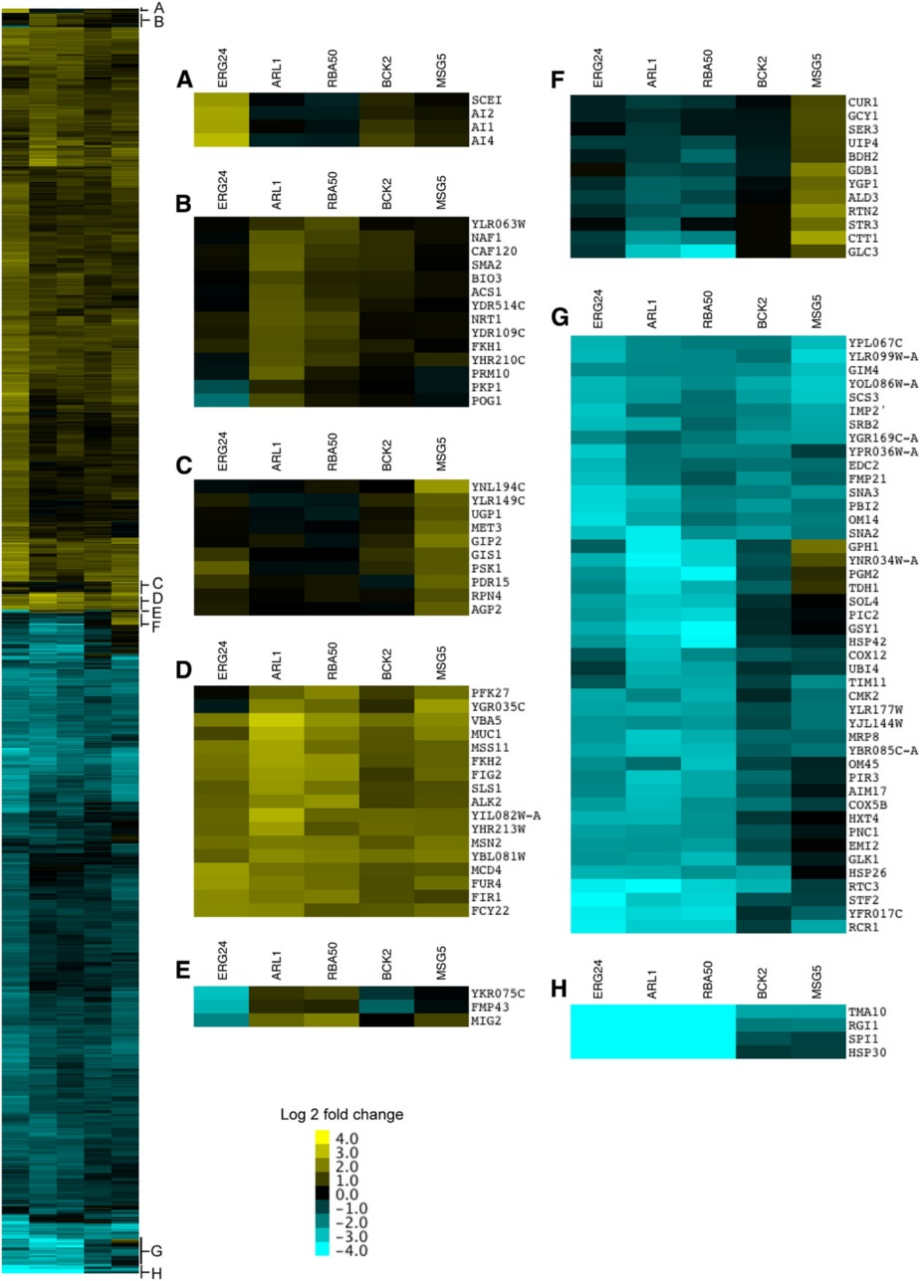
**Differentially expressed genes in COS-5.44 resistant yeast overexpressing strains in the presence of COS-5.44. **One thousand two hundred and twenty genes showed a significant change in expression in at least one of the overexpressing strains compared with the wild type (P-value ≤ 0.05 and log2 fold change ≥ 1 or ≤ −1). Clustering of the 1220 genes with significant change in expression was based in similarity (see methods). **A**–**D**) Subsets of up-regulated genes among the 5 overexpressing strains. **E**–**H**) Subsets of down-regulated genes among the 5 overexpressing strains.

Transcriptome analysis following COS-5.44 treatment was performed and 1220 genes (~589 up- and ~631 down-regulated) showed significant differential expression relative to the wild type under the same growth conditions (P-value < 0.05 and log2 fold change ≥ 1 or ≤ −1, Additional file 2: Table S4) in at least one of the 5 overexpressing strains (Figure 4). Significantly over represented biological processes identified by enrichment analyses [35,36] in the 589 differentially up-regulated genes included transcription, cell cycle, protein modification, stress response and RAS signal transduction (Figure 4A-E). Down-regulated genes were enriched for biological processes included protein folding, protein complex assembly, and respiratory chain complex genes (Figure 4F-H).

**Figure 5:**
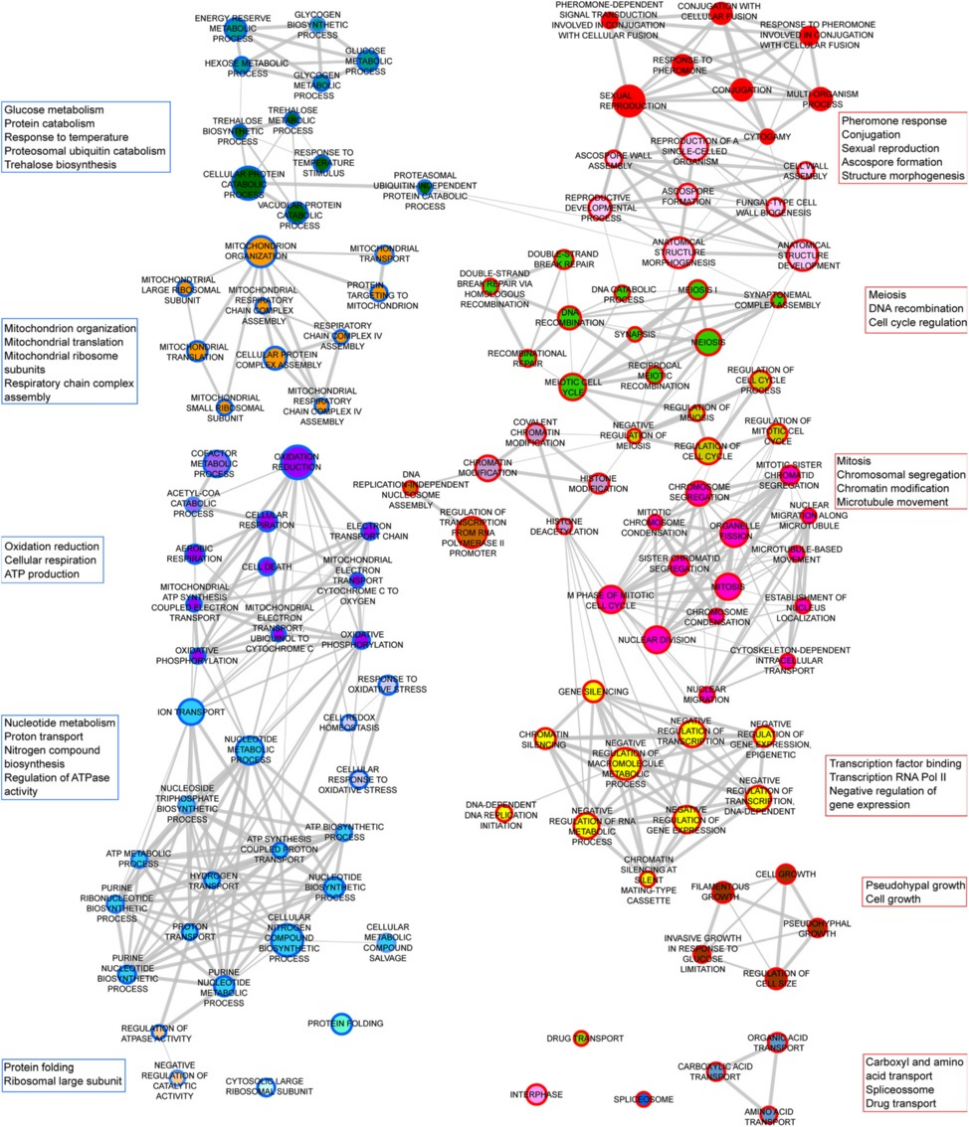
**Biological processes associated with differentially expressed genes on *Arl1 *overexpressing strain when exposed to chitosan oligosaccharide (COS).** A node represents a biological process significantly enriched (FDR ≤ 0.1, see Methods). Boxes on the side show summary of the main biological process found in a cluster. The node size correlates to the number of genes annotates to that functional category. Red and blue node border colours indicate enrichment of biological processes in up- and down-regulated genes, respectively. The width of the edge correlates to the degree of gene overlap between the 2 connected categories. If the overlap coefficient is less than 0.5, edges are not shown (see Methods). Cluster membership is shown by node color, where clustering is based in degree of overlap among categories.

The primary biological processes associated with the 5 overexpression strains in the presence of COS (Table 2) were: membrane signalling functions (*ARL1*, *BCK2*, *MSG5*), transcription (*RBA50*) and ergosterol synthesis (*ERG24*). To gain insight into the potential mechanisms of COS resistance for each overexpressed gene, enrichment maps were constructed from the entire transcription data set, GSEA analyzed, for each of the COS treated overexpressed strains (Figure 5 and Additional file 1: Figures S5- S9). Genes that were down-regulated to various degrees in all 5 strains were associated with processes such as cell energy generation (mitochondrial biology, ATP metabolism, energy storage metabolites) and associated by-products (oxidative stress). Most overexpressing strains (*ARL1*, *ERG24* and *RBA50*) displayed up-regulated genes involved in cell cycle progression (mitosis/meiosis, chromatin dynamics and modification and sporulation) and transcription. Taken together these results suggest that the overexpression resistant strains had an overall reduction in energy production and an increase in cell proliferation in response to COS-5.44 perturbation compared to wild type cells.

We confirmed some of the global transcriptional changes by qRT-PCR. *ARL1* overexpression was confirmed in *Arl1* overexpressing strains. For all of the genes selected, the qRT-PCR results were always in the same direction as the microarray results (i.e. increased transcript levels for a given gene found using microarrays was also found to be increased by qRT-PCR) but the dynamic range for the transcript changes was greater in the qRT-PCR assays. COS-5.44 treatment increased levels of expression in the *Arl1* overexpressing strain for *FIG2*, *MUC1*, *VBA5 and YJU2* with the qRT-PCR transcript levels being 50 - 200% higher when compared with the microarray data. Decreased transcript levels were confirmed for *CMD1*, *COX5B*, *HSP30, RCR1* and *UBI4*, using qRT-PCR (Additional file 1: Figure S10).

### *ARL1* overexpression reduces COS-induced membrane permeabilization

**Figure 6:**
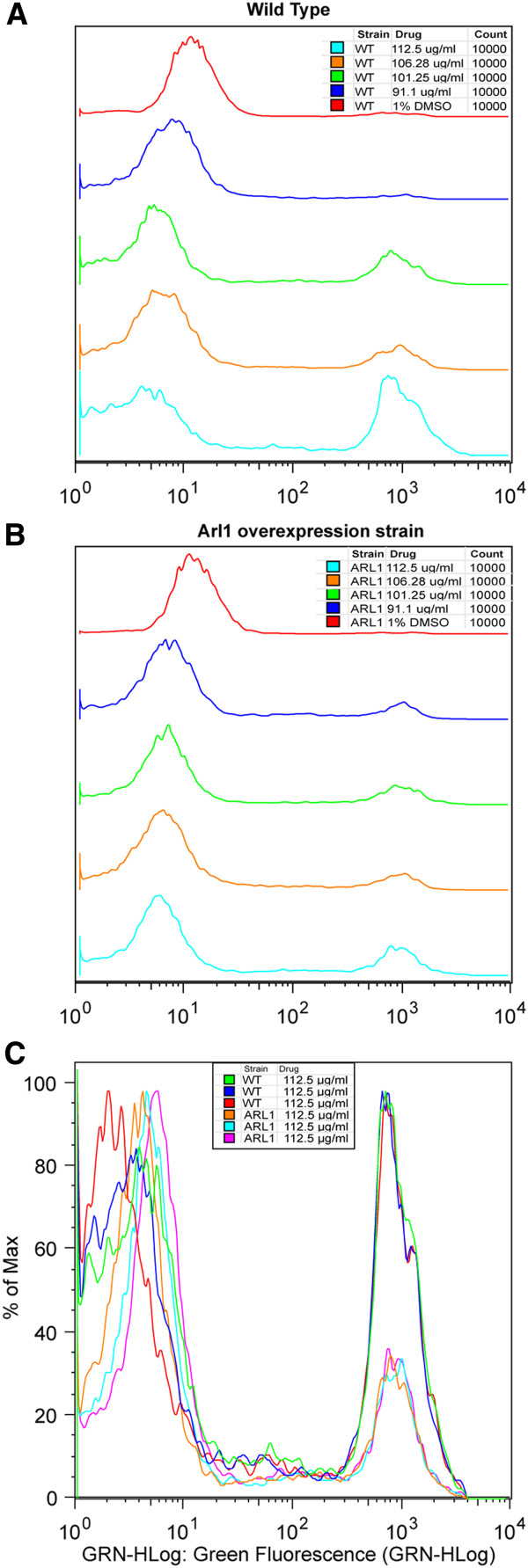
**Sytox cell permeability assay of COS-5.44 resistant *Arl1 *overexpressing strain vs. vector control (BY4743). A**) Vector control (BY4743) shows significantly higher cell membrane permeability compared with overexpressing strain *Arl1* (**B**) after treatment with chitosan oligosaccharide (COS-5.44). A large increase in cell membrane permeability is observed in the wild type as chitosan oligosaccharide concentration is increased (A). The first peak (left) in the graph corresponds to the background fluorescence and the second peak (right) corresponds to the Sytox signal observed inside the cell due to nucleic acid binding. **C**) Overlay of 3 replicates of each strain Sytox assay after exposure to112.5 μg/ml COS-5.44. Student *t*-test of C) data, P-value < 0.0101.

A Sytox permeability assay was performed in the wild type (BY4743) and *ARL1* strain after overnight growth in YPD with COS-5.44 to assess cell membrane permeability [37] see Methods. Sytox Green fluoresces once bound to nucleic acids. It only enters cells with compromised plasma membranes and is excluded from live cells with an intact plasma membrane. Wild type cells treated with COS-5.44 were Sytox-positive indicating that COS treatment causes membrane permeability (Figure 6A and 6C). There was a dose dependent increase in the intracellular Sytox fluorescence signal as a function of COS concentration (Figure 6A). Although there was some Sytox signal in the *Arl1* overexpressing strain, it was significantly lower than that in the wild type at the highest concentration of COS tested (112.5 μg/ml; P-value < 0.011, student t- test) (Figure 6C). This indicates that overexpression of *Arl1* provides protection against COS-induced cell permeability and damage.

### Yeast stress responses and COS-5.44 resistance

Given our observation that the transcriptional changes in the overexpressing strains include genes involved in stress responses, we investigated whether these strains had activated well known stress responses that could potentially account for the strain’s resistance to COS-5.44 treatment. To test this hypothesis, the wild type strain (BY4743) was exposed to a sub-lethal dose of different primary stresses previously reported by Berry and Gasch 2008, [38] followed by exposure to acute stress.

The wild type strain was first exposed to sub-lethal doses of stress (mild primary stress) followed by 2 h exposure to COS-5.44 (acute secondary stress). The primary mild stresses tested were: 0.25 M and 0.5 M NaCl, 1 M sorbitol, 0.003% and 0.006% H_2_O_2_ for 60 min, 37°C for 15 min and 30°C control (see Methods).

Wild type cells did not acquire resistance to COS-5.44 treatment after exposure to sub-lethal doses of different primary stressors (Additional file 1: Figure S11), although there was some variation in how the different primary stressors affected yeast growth when challenged with COS-5.44. In the 30°C control treatments, there was a similar amount of growth in both the control medium (0.5X YPD pH 5) and vehicle (1% DMSO); however, when COS-5.44 treatment was applied, we observed growth inhibition (Additional file 1: Figure S11A). After stressing the wild type cells for 15 min at 37°C, they could withstand 91.1 μg/ml of COS-5.44 and had a similar growth rate as wild type cells, but increasing COS concentrations dramatically inhibited growth rates (Additional file 1: Figure S11B). When the highest concentration of NaCl (0.5 M) was used as the primary stress, the effect of 112.5 μg/ml of COS-5.44 was increased, similar to what was observed when stressing cells for 15 min at 37°C (Additional file 1: Figure S11C). The addition of H_2_O_2_ slowed growth of the wild type cells and H_2_O_2_ pre-treatment did not appear to provide resistance to COS-5.44 (Additional file 1: Figure S11E-F).

In contrast with the other stressors, sorbitol (1 M) stress decreased growth in both untreated and COS-5.44 challenged cells (Additional file 1: Figure S11D). It should be noted that sorbitol primary stress does appear to provide some protection against the higher concentrations of COS with cells growing as well in the higher as the lower concentrations of COS, a property not observed for the other stressors. The osmotic stress generated by sorbitol treatment is known to activate other pathways such as MAPK and HOG cascades [39] and our results show that sorbitol pre-treatment provides some protection against COS (Additional file 1: Figure S11D)[39]. The sorbitol results are consistent with the findings of Zakrzewska et al. 2007, [23] who found that when HOG pathway mutants and wild type cells, were exposed to 1 M sorbitol, partial protection against chitosan was observed. Overall, these results suggest that the COS-5.44 stress response is different from the previously described environmental stress responses and as a result, most pre-stresses do not confer resistance to COS.

### *ARL1* resistance to other antifungal compounds or stresses

**Figure 7:**
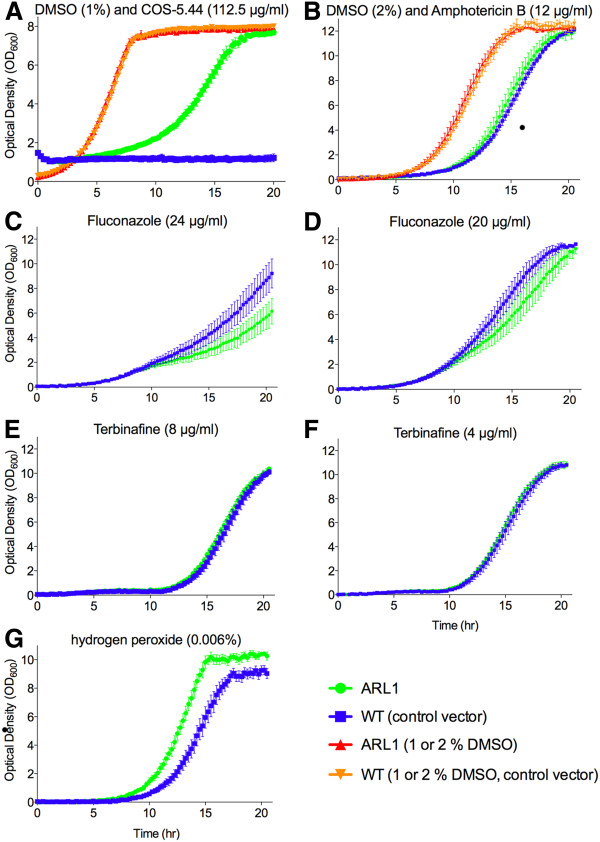
**Effect of osmotic stressors, antifungal agents and oxidative stress on the growth of *Arl1 *overexpressing and wild type cells. A**) *Arl1* overexpression confers resistance to COS-5.44 at a concentration that inhibits growth of the vector control cells. The *Arl1* overexpressing strain is as sensitive as the wild type cells to the tested antifungal compounds: **B**) Amphotericin B; **CD**) Fluconazole; **EF**) Terbinafine. Overexpression of *ARL1* confers tolerance to H_2_O_2_ (**G**) compared with the wild type. All assays were done in YPD except COS-5.44 that was done in 0.5X YPD. Optical density readings were taken every 15 min over 20 hrs using a Tecan Genios reader. Tecan ODs were converted to conventional 1 mm path length cuvette ODs using a calibration function provided by Ericson et al. 2010, [24]. Three colonies of each strain were grown in triplicate and compared with the wild type grown under the same conditions. YPD - Yeast Peptone Dextrose Broth.

To test whether overexpression of *ARL1* confers resistance to other antifungal agents, cationic compounds, or osmotic stresses, wild type (vector control) and *ARL1* overexpression strain cells were grown in the presence of these perturbations. Overexpression of *ARL1* did not confer resistance to NaCl, sorbitol or LiCl compared with the vector control (data not shown). Overexpression of *ARL1* does confer resistance to COS-5.44 at high concentrations (112.5 μg/ml) where the vector control is unable to grow (Figure 7A). Interestingly, the *ARL1* overexpression strain is as sensitive to Amphotericin B (12 μg/ml, Figure 7B) and Terbinafine (16 (not shown), 8 and 4 μg/ml, Figure 7E and F) as the wild type cells. The *ARL1* overexpression strain appears to be slightly more sensitive than the vector control to Fluconazole (32, 28, (not shown) 24 and 20 μg/ml, Figures 7C and D). These results suggest that the resistance to COS resulting from *ARL1* overexpression is specific and does not extend to other antifungal compounds that disrupt fungal cell membranes [40]. Overexpression of *ARL1* allows cells to withstand H_2_O_2_ perturbation slightly better (12%) than control cells suggesting that *ARL1* overexpression may be providing some protection against oxidative stress (Figure 7G).

### Synergistic interaction of COS-5.44 with fluconazole

**Figure 8:**
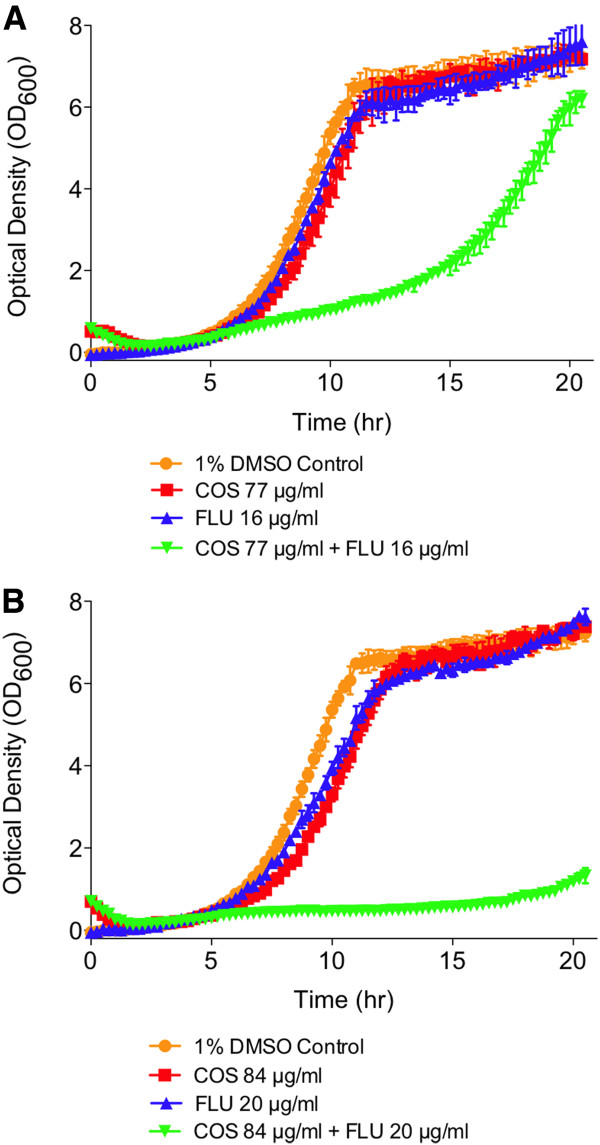
**COS-5.44 has a synergetic inhibitory effect on cell growth when used in combination with Fluconazole. A**) Growth curves of wild type strain (BY4743) growth in the vehicle, COS-5.44 (77 μg/ml), Fluconazole (16 μg/ml) and COS-5.44 + Fluconazole (77 + 16 μg/ml, respectively). **B**) Growth curves of wild type strain (BY4743) growth in the vehicle, COS-5.44 (84 μg/ml), Fluconazole (20 μg/ml) and COS-5.44 + Fluconazole (84 + 20 μg/ml, respectively). Optical density readings were taken as described in Figure 3.

Because COS-5.44 and Fluconazole have different modes of action, we examined the effect of treating cells with both compounds simultaneously to test if these two compounds might interact synergistically. We quantified the degree of interaction across different concentrations in a dose response matrix for each drug interacting with itself and with the other drug (Additional file 2: Table S8 - S11). At concentrations where either drug only weakly inhibits cells growth (i.e. COS inhibits growth ≤ 15% while Fluconazole inhibits ≤ 9%) when used in combination, a dramatic decrease in cell growth is observed (Figure 8A,B). Using the area under the growth curve as a metric, the combination of COS-5.44 and Fluconazole act in a synergistic fashion since the combination of both drugs inhibits growth much more (i.e. 45 to 86% inhibition) than the sum of growth inhibition caused by each compound individually.

## Discussion

We have applied multiple comprehensive chemogenomics assays in yeast to identify the gene targets of COS-5.44. Unlike other antifungal drugs tested with the same approach [24] where typically 3–10 genes are identified as potential drug targets, we found a much larger gene set (39) of yeast deletion strains highly sensitive to COS-5.44. The most sensitive heterozygous deletion strain in the pool will often be the drug target [24,26]. In our case, we identified 17 heterozygous deletion strains, suggesting that COS does not target a single specific protein as its antifungal mode of action. In a previous global fitness assay similar to the HIP-HOP assay, 101 homozygous strains were sensitive after 9 h (~ 6–7 generations) of exposure to 25 μg/ml of 10 kDa chitosan. Very few heterozygous strains were identified as sensitive to chitosan [23]. The differences seen in our results compared to those of Zakrzewska et al. [23] are likely due to a number of different experimental parameters. First, there were differences in the form and concentration of COS used, (i.e. a lower concentration and larger molecular weight of COS was used in that study [23]). As we described earlier, the physiochemical properties of COS such as degree of deacetylation and molecular weight are known to affect COS’s biological activity [16,41,42]. Second, the treatment period used in the Zakrzewska et al. study was shorter than in the one used in our study (9 vs. 40 hrs equivalent to ~ 6–7 and 20 generations respectively). The shorter exposure time could explain the low number of chitosan sensitive heterozygous deletion strains found by these authors since the subtle phenotypes of the heterozygous deletions strains often require longer growth periods to resolve fitness differences between strains [43]. Finally, the lower threshold for the identification of sensitive strains (i.e. those having a log2 ratio of  ≥ 1.585) used by Zakrzewska et al. 2007, [23] accounts for the higher number of homozygous strains they determined to be chitosan sensitive.

Several of the genes sensitive to COS in our HIP-HOP assay and resistant in the multicopy suppression assay were enriched for proteins targeted to membranes. This is not surprising given that COS likely perturbs membrane integrity. COS might also be creating oxidative stress leading to the accumulation of damaged proteins that are degraded by the proteosome. Protein degradation/proteosome functions were among the biological processes enriched in the HIP-HOP assays (Figure 2C).

**Figure 9:**
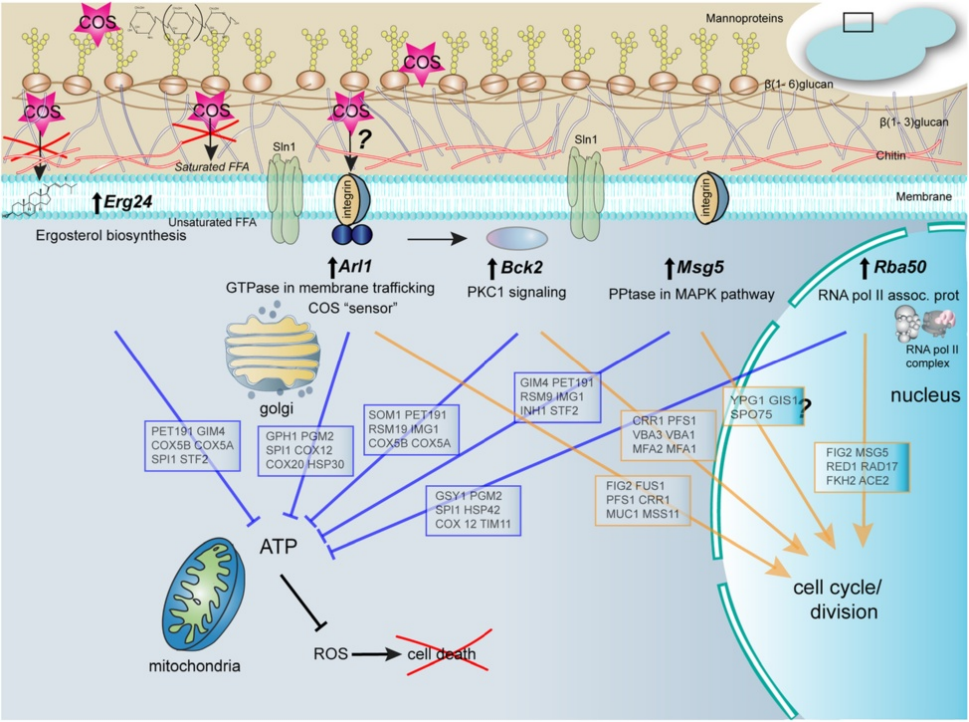
**Potential mechanisms of COS resistance resulting from the overexpression of specific genes identified in this study. **In this model, *ERG24* overexpression would reduce membrane permeability by altering the composition and fluidity of the plasma membrane. *ARL1*, *BCK2*, *MSG5* and *RBA50* either alone or in combination, could detect COS binding to the membrane and subsequently induce transcriptional and additional changes that reduce membrane permeability. Overexpressing these strains that are resistant to COS results in a down regulation of transcripts related to energy production (e.g. ATP synthesis and mitochondrial activity; examples of specific down-regulated genes are listed in the blue boxes). A large number of genes involved in cell division (mitosis and meiosis), cell cycle and cytoskeleton are transcriptionally up-regulated in the COS resistant overexpression strains (examples listed in the orange boxes). Integrin refers to yeast proteins that have properties similar to a mammalian integrins.

We selected 5 of the confirmed overexpressing strains that provide resistance to COS to uncover any changes in their gene expression profiles that might give insights into COS’s mechanism of action. *ARL1* (YBR164C) was found to be sensitive to COS as a deletion strain in the HOP assay and resistant in the MSP assay in this study and was found to be sensitive to chitosan as a homozygous deletion strain in a previous study [23]. We confirmed that an *ARL1* deletion strain was sensitive to COS-5.44 and overexpression of *ARL1* conferred resistance to COS-5.44 (Figures 3B and 3D). We believe the identification of this gene does provide information into the molecular mechanism of COS-5.44. *Arl1* is a G protein and soluble GTPase and is a member of the Ras superfamily [28,44]. *Arl1* is highly conserved in all eukaryotes with 65% homology to human ADP-ribosylation factor-like protein 1 (Arl1). *Arl1* has been shown to be associated with the *trans*-Golgi and is thought to be required for endosome-Golgi trafficking [45,46]. *Arl1-*GTP recruits specific receptor proteins to the membrane surface by binding to their GRIP domains, although in yeast, no specific *Arl1* binding receptor proteins have yet been identified [44,45]. Mutations in yeast *ARL1* are not lethal [28] but *ARL1* mutants do show mild defects in localizing proteins to vacuoles as well as defects in potassium uptake [44,46-48]. *arl1* mutants have also been shown to be more sensitive to antifungal agents such as Hygromycin B [46]. *Arl1* can be myristolayted and we hypothesize that through myristoylation, the soluble form of the GTPase could be bound to the membrane bilayer. In this way *ARL1* could act as a sensor and modulate membrane trafficking at the onset of COS 5.44 induced membrane permeabilization (Figure 9). *Arl1* (*Arl1p*, PDB id 1moz) has been predicted to weakly associate with membranes (deltaG of -4 kcal/mol without ligands for the dimer) with the N-terminal residues (N-myristoyl glycine) of each dimer binding to the membrane [27,49,50]. GTPases are known to be involved in signal transduction pathways in filamentous fungi and yeast. These proteins are also involved in conidiation, a process that is enhanced by chitosan treatment in filamentous fungi [8]. When the *ARL1* gene is overexpressed and yeast cells are challenged with COS, a large set of genes related to cytoskeleton organisation (e.g. microtubule dynamics) and stress sensing are up-regulated (Figure 5). These genes are also involved in cell division (mitosis and meiosis) and the cell cycle. This could explain the ability of chitosan to enhance sporulation [28,44] .

*ERG24*, which encodes C-14 sterol reductase, is a gene involved in ergosterol biosynthesis. Because ergosterol is a unique lipid in fungal membranes it is a classical target of many antifungal compounds. In a previous study, while ergosterol content was not associated with resistance to chitosan, in some filamentous fungi saturation of free fatty acids was associated with resistance [51]. Free fatty acid desaturases lower membrane fluidity and desaturase mutants have been found to confer resistance to chitosan in *N. crassa*[51]. Membrane patches rich in ergosterol and other lipids alter membrane fluidity and are important for correct membrane function [52]. It also has been shown that an increase in sterol biosynthetic capacity results in an increase in the availability of fatty acids [53]. We can envision that overexpression of *ERG24*, a key gene in the ergosterol biosynthetic pathway, could increase the size/presence of ergosterol rich membrane rafts and fatty acid content making the membrane less fluid and therefore more resistant to COS.

For both yeast and filamentous fungi, the plasma membrane is the primary target for chitosan. In yeast, chitosan causes cell leakage and stops cell growth [22]. In filamentous fungi, chitosan permeabilizes the membrane in an energy dependent manner and kills cells of sensitive species such as *N. crassa* conidia [54]. A recent study by Palma-Guerrero et al. 2010, [51] found that membrane fluidity is a key factor in the antifungal action of chitosan with fluid fungal membranes binding more chitosan than less fluid ones. Chitosan crosses the fungal cell wall and binds to membrane lipids with a preference for negatively charged ones [51]. Chitosan permeabilizes the membrane when the concentration reaches a critical level [55,56]. Our study identifies pathways involved in membrane structure and signalling that are affected by COS. The Sytox uptake experiments confirm that membrane permeability is increased when cells are exposed to COS and that overexpression of *ARL1* suppresses the increase in membrane permeability caused by COS (Figure 6).

While our work and previous studies have shown that chitosan induces stress responses in yeast and other fungi, our study shows that pre-treatment with any of the well characterized environmental stresses (thermal, salt, osmotic and oxidative) does not provide resistance to COS-5.44 in yeast (Additional file 1: Figure S11). Therefore, the changes induced by COS would appear to be different from previously described environmental stress response pathways.

Assuming that COS’s main mode of anti-fungal action is to increase the permeabilization of membranes, we speculate that several of the genes we have identified in this study are involved in a cellular response that reduces permeabilization of the membrane and/or mitigates the damage induced by the permeabilization. For example, *ARL1*, *BCK2* and *MSG5* could be part of a sensing and signalling mechanism(s) that detects COS binding to the membrane that induces transcriptional and other changes to reduce membrane permeability. *ERG24* could be reducing membrane permeability by affecting the composition and fluidity of the membrane (Figure 9). Ultimately, if COS concentrations are too high, the cellular response would be insufficient to prevent membrane permeabilization and cell death would ensue - possibly as a result of the release/leakage of oxygen radicals from the mitochondria. While the exact mechanism of cell death by COS is still unknown, it appears to be a respiration/ATP-dependent process in filamentous fungi [54]. Overexpressing strains resistant to COS display down-regulated genes related to energy dependent processes (ATP synthesis, mitochondrial activity, etc.) (Figure 5; Additional file 1: Figures S5-S9; Additional file 2: Table S6). In this sense, the overexpressing strains would mimic the effect of treatments such as sodium azide and low temperature that inhibit oxidative respiration and chitosan damage to fungal cells [54]. Interestingly, the *ARL1* overexpression strain also shows increased resistance to certain forms of oxidative stress such as H_2_O_2_ (Figure 7G). The transcriptome analysis of several of the overexpressing strains suggests that resistance to COS involves generation of less ROS by reducing oxidative respiration as well as reducing cell responses to ROS.

A comparison of the gene targets found in the HIP-HOP screens in this study to genes found in similar screens for other fungicides that act at the cell membrane (e.g. Amphotericin B, Fluconazole and Terbinafine) suggests that COS may have a different mechanism of action than these other drugs [57]. In support of this hypothesis, we have found that the *ARL1* overexpression strain does not provide resistance against Amphotericin B, Fluconazole or Terbinafine (Figure 7B-F). This suggests that COS might be an effective antifungal agent in strains that are resistant to the other perturbing cell membrane compounds.

Fluconazole, Terbinafine and Amphotericin B antifungal activity involves the targeting of ergosterol, the principal cell membrane sterol of fungi, by different mechanisms. Fluconazole (an triazole) inhibits 14α-demethylase (lanosterol demethylase, *Erg11*), a fungal cytochrome P450 dependent enzyme, and depletes cell membrane ergosterol resulting in reduced membrane fluidity, and the accumulation of 14α-demethylated sterols that are toxic to the cell. This leads to growth arrest and eventual fungal cell death [58,59]. Terbinafine inhibits squalene monoxygenase (*Erg1*), an enzyme responsible for conversion of squalene epoxide, a precursor to lanosterol in the ergosterol synthesis pathway [59]. Amphotericin B targets the fungal cell membrane by directly binding to ergosterol, forming complexes that intercalate the cell membrane, resulting in formation of pores and leakage of intracellular contents [60,61]. COS appears to perturb the cell membrane possibly through the ergosterol pathway, by targeting *ERG24*, a gene that encodes for C-14 sterol reductase [62].

Because COS seems to have a distinct mode of action compared with other antifungals, we were interested in seeing its inhibitory effect when used in conjunction with another antifungal drug. We tested COS and Fluconazole in combination (as well as on their own), and saw a strong synergism between them. That is, at concentrations where either drug alone had little inhibitory effect, a strong inhibitory effect that was much greater than additive was observed when they were combined (45 to 86% growth inhibition; Figure 8A,B). One possible explanation for this synergy might be that the increased membrane permeability induced by COS allows more Fluconazole to penetrate the cell membrane to inhibit 14α-demethylase that in turn further reduces membrane fluidity and increases the production of toxic sterols.

## Conclusions

The results of this study have provided insights into the molecular mechanisms of the antifungal modes of action of COS. Some of the pathways COS appears to affect include sensing, signalling, and the composition of the cell membrane (Figure 9). Our results suggest that COS does not have a single specific gene target. We provide evidence that COS increases cell membrane permeability and that the overexpression of *ARL1* can reduce the permeability induced by COS. Resistance to COS does not confer resistance to other antifungal compounds that disrupt the fungal cell membrane such as azoles, polyenes and Terbinafine. COS can synergize with other antifungal agents such as Fluconazole suggesting that COS could be considered as alternative antifungal treatment to fungal pathogens that are resistant to other antifungal compounds or be used in combination with other antifungal agents to enhance their activity.

## Methods

### Chitosan oligosaccharide

Chitosan oligosaccharide (COS-5.44) (5.44 KDa, PDI (poly dispersity index) = 1.14 and 97% degree of deacetylation) was kindly provided by Dr V. Tikhonov (Laboratory of Physiologically Active Biopolymers, A. N. Nesmeyanov Institute of Organoelement Compounds, Russian Academy of Sciences, Moscow, Russia). COS-5.44 was prepared as described by Tikhonov et al. 2006, [20]. For the experiments performed in this study, a COS-5.44 stock solution (25 mg/ml) in 50% DMSO was prepared, aliquoted and stored at −20°C.

### Chitosan

Chitosan (T8s) with a molecular weight of 70 kDa and 80% degree of deacetylation was obtained from Marine BioProducts GmbH (Bremerhaven, Germany). Chitosan was prepared as described by Palma-Guerrero et al. 2008, [55]. Each batch of dialyzed and autoclaved chitosan was stored at 4°C for a maximum of 2 weeks.

### Yeast sensitivity to COS-5.44 and chitosan

A pre-screen of COS-5.44 and chitosan (T8s) in wild type yeast (BY4743) was performed as described in Pierce et al. 2007, [43] to determine the antifungal activities of these two compounds that have different molecular weights and degrees of deacetylation. For the pre-screen of COS-5.44, concentrations from 62 μg/ml to 125 μg/ml of COS-5.44 were tested (Figure 2B). 98 μl of cell mix at an initial OD_600_ of 0.0625 in 0.5X YPD pH 5 (5 g bacto yeast extract, 10 bacto peptone and 10 g dextrose in 1 l distilled water [63]) was added to each well of a 96 well plate (Nunc) then 2 μl of the corresponding COS-5.44 stock were added, including carrier controls (50% DMSO and 0.25 mol/l HCl neutralized pH5.6). The chitosan (T8s) screen followed the same protocol, with the slight difference in volume of cell mix (97 μl) added and chitosan (T8s) stock (3 μl). Concentrations from 125 μg/ml to 162.5 μg/ml of chitosan (T8s) were tested (Additional file 1: Figure S1) Cells were grown at 30°C in a shaking-spectrophotometer (Tecan), with readings taken every 15 min for 24 hrs. Tecan ODs were converted to conventional 1 mm path length cuvette ODs using a calibration function provided by Ericson et al. 2010, [24]. The COS-5.44 pre-screen was performed to identify an IC_70-90_ and IC_10-20_ of COS-5.44 in the BY4743 to be used in the multicopy suppression and deletion profiling (HIP-HOP) respectively (Figure 2B).

### Haploinsufficiency and homozygous profiling (HIP-HOP) assay

Haploinsufficiency and Homozygous profile assays, genomic DNA purification, PCR amplification of barcodes, array hybridization, analysis and confirmation were performed as described in Ericson et al. 2010, [24]. The homozygous collection was grown for 5 generations (approximately 10 hrs) and the heterozygous collection for 20 generations (approximately 40 hrs), as these periods of time have been found to be optimal to allow each collection to resolve growth differences [43].

Experiments were performed in duplicate. Yeast deletion strains with log2 ratio of 3.5 or higher (Highly sensitive COS-5.44 deletion strains) were selected for individual confirmation. A log2 ratio of 3.5 represents an approximately 11- fold less abundance of the deletion strain after COS treatment.

### Multicopy suppression profiling and screen conditions

MSP screen, plasmid isolation, Affymetrix TAG4 array hybridization, microarray results analysis and confirmation followed the protocols described by Hoon et al. 2008, [25] and Ericson et al. 2010, [24].

### Transformation of candidate resistant genes for overexpression in yeast

Sixty-eight genes were identified with MSP as possible candidates to confer resistance to COS-5.44 (Additional file 1: Figure S2). To confirm which of the 68 genes conferred resistance, each gene was individually overexpressed in yeast. For this purpose *E. coli* strains carrying the genes of interest were obtained from the MoBY-ORF 2 μ collection, kindly provided by Sarah Barker from the Boone lab [30]. 57 of the 68 candidate genes were available in the MoBY-ORF 2 μ collection and thus 11 of the genes of interest were not present in this collection and could not be tested.

Individual colonies of each *E. coli* strain were grown in selective media in 2YT (1% Yeast extract, 1.6% Bacto tryptone, and 0.5% Sodium) + 0.4% Glucose + Carbenicillin (200 mg/ml, SIGMA) + Kanamycin (50 mg/ml, SIGMA) in 96 square well blocks (Greiner, Bio-one) at 37°C and 200–250 rpm. Cells were harvested (5 min, 4000 rpm) to proceed with DNA extraction. Macherey-Nagel Multi-96 purification kit (MN Cat # 740 625.4) was used to miniprep the plasmids, following the manufacturer’s protocol. Elution was done in 150 μl to collect ~ 100 μl of purified DNA.

Each plasmid extract was double digested with EcoRI/XhoI (New England BioLabs Inc.). Digested samples were run on a 0.8% agarose gel to confirm vector and ORF fragments size.

The confirmed plasmids were transformed into yeast strain BY4743 (*his3 1/his3 1 leu2 0/leu2 0 ura3 0/ura3 0 met15 0/+ lys2 0/+*), using a 96 well yeast transformation protocol as described by Gietz et al. 2007, [64]. Five single colonies of each strain were grown overnight in selective liquid media then archived in 15% glycerol at −80°C.

Once overexpressing strains were obtained, confirmation of resistance to COS-5.44 was performed (Figure 1). Overexpressing strains were grown at different concentrations of COS-5.44 previously found to be inhibitory to the wild type strain in MSP. Concentrations tested were 101.25, 106.28, 112.5, 118.75, 125.0 and 250.0 μg/ml. To ensure that the resistance was conferred by the overexpressed gene, plasmid DNA was extracted from two colonies of each overexpressing strains, and transformed into *E. coli*. Amplified plasmids were extracted using Genejet kit (Fermentas) following manufacturer protocol. Purified plasmids were double digested as described. Confirmed plasmids were retransformed into BY4743 and **t**hree single colonies were selected and confirmed for COS-5.44 resistance as previously described.

### Sample collection and RNA extraction of overexpressing strains

A confirmed colony of each overexpressing strain and the wild type with the empty vector was grown overnight at 30°C and shaking at 200–250 rpm. An overnight culture was used to inoculate 3 flasks each with 400 ml of 0.5X YPD pH 5. Cells were grown until the culture reached an OD_600_ of 0.8 and a 100 ml sample was collected (time zero). The remaining 300 ml were treated with COS-5.44 (112.5 μg/ml) and 100 ml samples were collected at 15, 30 and 60 min after treatment (Figure 1). Cells were collected by centrifugation (5 min, 4000 rpm) frozen with liquid nitrogen and stored at −80°C, for RNA extraction and labeling. Once all samples were collected a hot acid phenol-chloroform RNA extraction was performed [65].

Microarray analysis (see below) of the COS treated wild type cells found that the maximum number of differentially expressed genes were obtained with the 60 min treatment with minimal loss of genes differentially expressed at earlier time points but not at 60 min. Therefore, for the overexpression strains, microarray analysis was only performed on the untreated and 60 min COS treated samples.

### cDNA and microarray sample preparation

cDNA synthesis and sample labeling for NimbleGen 4X72k yeast microarrays (Roche NimbleGen, Inc.; Design ID A6186-00-01, TI4932 60mer expr X4) was performed as described in the manufacturer’s protocol with minor modifications. cDNA synthesis was performed using 10 μg of total RNA. The sample labeling reactions were done using 1 μg of double stranded cDNA. Three cDNA biological replicates either with or without a 60 min exposure to COS-5.44 for each of the 5 overexpressing strains as well as an untransformed wild type BY4743 cells (vector control; for a total of 36 samples) were hybridized to NimbleGen 4X72k microarrays (Roche NimbleGen, Inc.). Microarrays were scanned with a Genepix 4000B scanner (Molecular Devices Inc. Sunnyvale, CA).

### Microarray data analysis

Raw images were obtained using GenePix Pro software (Version 5.0, Molecular Devices Inc. Sunnyvale, CA). Raw probe intensities (x, y and signal reports) were obtained using NimbleScan software (v2.4, Roche NimbleGen, Inc). Data preprocessing of 60mer oligonucleotide arrays was performed using the BioConductor package Oligo [66]. Genes with significant expression difference compared with the vector control (wild type, BY4743) were identified for each overexpressing strain using the BioConductor package Limma Linear models for microarray analysis, [67], taking P-value ≤ 0.05 and log2 fold change of  ≥ 1 or  ≤ −1 as significant unless otherwise noted. The data discussed in this publication have been deposited in NCBI’s Gene Expression Omnibus [68] and are accessible through GEO Series accession number GSE32888 (http://www.ncbi.nlm.nih.gov/geo/query/acc.cgi?acc=GSE32888).

### Hierarchical clustering

Hierarchical clustering of the data was performed using the program Cluster 3.0 originally written by Eisen, [69], modified by de Hoon, [70]. Expression data of the 5 overexpressing strains was clustered using city-block distance measurement and average linkage clustering method. The resulting clusters were visualized using Java TreeView [71].

### Functional annotation of chemogenomic assays and microarray results

The genome-wide profile of the COS-5.44 sensitive deletion strains was examined using Gene Set Enrichment Analysis GSEA, [29] to identify enriched biological processes. GSEA has been considered advantageous for quantitative genome-wide profiles because it can take into account the entire profile, exploiting the weighting of the genes (e.g. degree of sensitivity to a drug (i.e. fitness defect), differential gene expression). Other functional analysis methods focus on a list of genes that satisfy the characteristic of interest. This would first require setting a threshold to divide the profile into lists of genes that satisfy the characteristic of interest and those that do not. It is often unclear how to choose an appropriate threshold, yet with GSEA, choosing such a threshold is not required.

Deletion strains were mapped to genes using chromosomal feature data downloaded from the *Saccharomyces* Genome Database (SGD) on April 16, 2011. Multidrug resistance genes MDR, [57] were filtered from the genome-wide profile of sensitive deletions strains to COS, to identify biological process specific to COS response. The list of all genes ranked by their fitness defects was analyzed by GSEA v2.07 (4864 genes, without MDR genes) [29]. Default parameters were used except that minimum and maximum gene set size were restricted to 5 and 300, respectively. Biological process annotation was obtained from Gene Ontology website (http://berkeleybop.org/goose) on April 13, 2011. Additional protein complex annotations based on consensus across different studies were obtained from Benschop et al. 2010, [72].

Enrichment maps were generated with Enrichment Map Plugin v1.1 [73] developed for Cytoscape [74] with default parameters. Nodes in the maps were clustered with the Markov clustering algorithm, using an overlap coefficient computed by the plugin as the similarity metric (coefficient < 0.5 were set to zero) and an inflation parameter with value of 2. For each cluster leading edge were computed as in Subramanian et al. 2005, [29] for each member of a node. The top 10 leading edge genes associated with the most nodes in the cluster are shown in the bar plot (if less than 10 genes, all are shown).

A functional analysis (i.e. GSEA [29]) was performed as described above for the transcriptional profiles of the wild type (BY4743) exposed to COS-5.44 and the 60 min chitosan data set from Zakrzewska et al. 2005, [22] that was performed on X2180-1A, *MAT*a *SUC2 mal gal2CUP* cells. The set of environmental stress response genes ESR, [75], were filtered from the transcriptome data sets to identify the biological processes enriched by COS or chitosan treatment apart of the general stress response of yeast. Enrichment maps were generated with Enrichment Map Plugin v1.1 [73] developed for Cytoscape [74] with the following parameters: FDR < 1%, p-value < 0.005 and using an overlap coefficient computed by the plugin as the similarity metric (coefficient < 0.5 were set to zero). Enrichment maps were compared with the comparison function of the Enrichment Map Plugin v1.1 [73].

A similar functional analysis was performed on the whole transcriptome dataset of each overexpressing strain. As was described above, the set of environmental stress response genes were filtered from the transcriptome data set. An additional enrichment analysis of significantly up- or down-regulated transcripts (P-value ≥ 0.05 and log2 fold changes ≥ 1 or ≤ −1) was also performed using the single enrichment analysis tool of Babelomics [35,36]. All default parameters were used.

### Quantitative RT-PCR

Quantitative reverse transcription – PCR (qRT-PCR) verification of the microarray results for selected genes, was performed using KAPA SYBR FAST (KAPA Biosystems) in a CFX384 real-time PCR detection system (Bio-Rad), with two biological replicates from wild type (vector control BY4743) or *Arl1* overexpressing strain that were either untreated or exposed to COS-5.44 for 60 min. Each RT qPCR reaction had 200 pg of cDNA, 5 μL of KAPA SYBR FAST master mix (2x), 300 μM of each primer and water to have a final volume of 10 μL per reaction. Cycling conditions used were as follows: 95°C for 3 min, followed by 40 cycles of 95°C for 3 s and 60°C for 30 s then 95°C for 10 s. All the reactions were performed in triplicate, using primers for the following genes: *ACT1*, *ARL1*, *CMD1*, *COX5B*, *ERV25*, *FIG2*, *HSP30*, *MUC1*, *RCR1*, *RPL32*, *SPT15*, *UBI4* and *VBA5*. Primers were designed using Primer-BLAST Developed at NCBI uses Primer 3, [76]. See Additional file 2: Table S6 for the sequences of the primers used. The average of three technical replicates was normalized to four internal control transcripts, *ACT1*, *ERV25*, *RPL32*, *SPT15*, there were not significant differences among internal controls therefore all were used for normalization. Data was analyzed using CFX Manager (Bio-Rad laboratories, Inc).

### Cell permeability assay

Plasma membrane permeability was measured by SYTOX Green uptake as described by Thevissen et al. 1999, [37] with modifications. *S. cerevisiae* cells of the wild type and overexpressing strain *Arl1* were grown in the presence of COS-5.44 (91.1, 101.25, 106.28 and 112.5 mg/ml) and vehicle control (1% DMSO). Cultures of the overexpressing strain *Arl1* and wild type (B4743) with empty vector were set up at initial OD of 0.0625 and grown overnight (17 hrs). After COS-5.44 treatment the cells were pelleted by centrifugation (5 min, 4000 rpm) and washed three times in 0.1 M Tris–HCl, pH 7.0. Two hundred microlitre aliquots of the yeast cell suspension was incubated with 0.2 μM SYTOX Green in 96-well microplates for 30 min at 30°C with periodic agitation in the dark. Three replicates of each strain were performed. Fluorescence was measured using a Guava easyCyte flow cytometer (Millipore). The green fluorescence filter set 488 nm (excitation) and 520 nm, (emission) was used. Fluorescence data was analyzed with the software FlowJo (V9.3.1, Tree Star, Inc.).

### Stress response and COS-5.44 resistance

To verify if the activation of the environmental stress response (ESR) provides resistance to COS-5.44 treatment, the wild type strain (BY4743) was exposed to sub-lethal doses of primary stresses previously reported by Berry and Gasch 2008, [38] to provide resistance to more severe stresses. The yeast wild type strain was exposed to sub-lethal doses of stress (mild primary stress) then cells were exposed to chitosan oligosaccharide treatment (severe secondary stress). The primary mild stresses tested were: 0.25 M and 0.5 M NaCl, 1 M sorbitol, 0.003% and 0.006% H_2_O_2_ for 60 min, 37°C for 15 min and 30°C control. Yeast cells were collected by centrifugation (5 min, 4000 rpm) then exposed to the secondary stress treatment COS-5.44 for two hours (91.1, 101.25, 0.1068 and 112.5 μg/ml). After the secondary stress, cells were collected by centrifugation, transferred to YPD and their growth rates measured.

### Resistance to other antifungal and cationic compounds

To ask if the overexpression of *ARL1* conferred resistance to other cationic compounds, the wild type and *ARL1* overexpression strain were grown in the presence of other cationic compounds (as described in section 3.2).

A dose response assay was performed in the wild type and overexpression strain *ARL1*, as previously described screen protocol, using the following compounds: NaCl (0.5 and 1 M); sorbitol (1 and 2 M); LiCl (0.01 M); hygromycin B (25, 12, and 6 μg/ml); Amphotericin B (25, 12 and 6 μg/ml); Fluconazole (32, 28, 24 and 20 μg/ml); Terbinafine (16, 8 and 4 μg/ml); H_2_O_2_ (0.012% 0.006%); 1% SDS (0.05, 0.025 and 0.012%).

### Interaction between COS and fluconazole

For COS-5.44, a concentration range of 35–105 μg/ml and for Fluconazole, a concentration range 4–24 μg/ml was used. Stocks for each drug treatment were prepared in 12.5% DMSO resulting in a final concentration of 1% DMSO. Wild type yeast (BY4743) was grown overnight and diluted at an initial OD_600_ of 0.0625 in 0.54X YPD pH 5. 92 μl of cell mix was aliquoted to each well of a 96 well plate (Nunc), and 4 μl of the corresponding COS-5.44 and/or Fluconazole stock were added in a 7 × 12 dose response matrix. Two dose response matrices were performed: self-self (COS-5.44 – COS-5.44 and Fluconazole – Fluconazole) and two replicates of the COS-5.44 and Fluconazole matrix. Cells were grown at 30°C in a shaking-spectrophotometer (Tecan), with readings taken every 15 min for 24 hrs. We used the area under the growth curve as a metric to measure growth inhibition and drug interaction.

## Abbreviations

COS: Chitosan oligosaccharide or chitooligosaccharide; DMSO: Dimethyl sulfoxide; ESR: Environmental stress response; FDR: False discovery rate; FLU: Fluconazole; GEO: Gene expression omnibus; GSEA: Gen set enrichment analysis; HIP: Haploinsufficiency profiling; HOG: High osmolarity glycerol; HOP: Homozygous profiling; IC: Inhibitory concentration; Limma: Linear models for microarray analysis; MAPK: Mitogen-activated protein kinase; MDR: Multidrug resistance genes; MoBY-ORF: Molecular barcoded yeast ORF library; MSP: Multicopy suppression profile; PDI: Poly dispersity index; qRT-PCR: Quantitative reverse transcription – PCR; Ras superfamily: Rat sarcoma superfamily; ROS: Reactive oxygen species; SGD: *Saccharomyces* genome database; YPD: Yeast peptone dextrose; YT: Yeast triptone.

## Competing interests

The authors declare that they have no competing interests.

## Authors’ contributions

MDLAJ, LVLL, JTW and CN conceived, designed the experiments and wrote the manuscript. GG provided the chemogenomic assay platform and reagents. MDLAJ and MG performed chemogenomic assays. MDLAJ carried out the overexpression strains study. MDLAJ, AYL and LEH performed chemogenomics data analysis and enrichment maps. MDLAJ and MP performed analysis of yeast strains growth confirmations. MDLAJ and AC carried out the transcriptional analysis. AC, MP, LEH provided advice in data analysis. Experimental work was carried out in the labs of GG, JTW, CN and the Canadian Drosophila Microarray Center (CDMC). All authors read and approved the final manuscript.

## Supplementary Material

Additional file 1 Figures S1 – S11 and their corresponding figure legends. Click here for file

Additional file 2 Tables S1 – S13 and their corresponding legends. Click here for file

## References

[B1] Kumar M A review of chitin and chitosan applications React Funct Polym 2000 46 1 27

[B2] Hayes M Carney B Slater J Bruck W Mining marine shellfish wastes for bioactive molecules: chitin and chitosan–Part A: extraction methods Biotechnol J 2008 3 871 877 18320562 10.1002/biot.200700197

[B3] Bartnicki-Garcia S Cell wall chemistry, morphogenesis, and taxonomy of fungi Annu Rev Microbiol 1968 22 87 108 4879523 10.1146/annurev.mi.22.100168.000511

[B4] Ruiz-Herrera J Gonzalez-Prieto JM Ruiz-Medrano R Evolution and phylogenetic relationships of chitin synthases from yeasts and fungi FEMS Yeast Res 2002 1 247 256 12702327 10.1111/j.1567-1364.2002.tb00042.x

[B5] Kurita K Chitin and chitosan: functional biopolymers from marine crustaceans Mar Biotechnol (NY) 2006 8 203 226 16532368 10.1007/s10126-005-0097-5

[B6] Kim SK Rajapakse N Enzymatic production and biological activities of chitosan oligosaccharides (COS): A review Carbohyd Polym 2005 62 357 368

[B7] Allan CR Hadwiger LA The fungicidal effect of chitosan on fungi of varying cell wall composition Exp Mycol 1979 3 285 287

[B8] Palma-Guerrero J Larriba E Güerri-Agulló B Jansson H-B Salinas J Lopez-Llorca LV Chitosan increases conidiation in fungal pathogens of invertebrates Appl Microbiol Biot 2010 87 2237 2245 10.1007/s00253-010-2693-1 20532757

[B9] Hirano S Nagao N Effects of chitosan, pectic acid, lysozyme, and chitinase on the growth of several phytopathogens Agric Biol Chem 1989 53 2

[B10] El Ghaouth A Arul J Asselin A Benhamou N Antifungal activity of chitosan on post-harvest pathogens: induction of morphological and cytological alterations in Mycol Res 1992 769

[B11] Benhamou N Lafontaine PJ Nicole M Induction of systemic resistance to Fusarium crown and root rot in tomato plants by seed treatment with chitosan Phytopathology 1994 84 1432 1444

[B12] Laflamme P Benhamou N Bussieres G Dessureault M Differential effect of chitosan on root rot fungal pathogens in forest nurseries Can J Bot 1999 77 1460 1468

[B13] Hadwiger LA Beckman JM Chitosan as a component of pea-Fusarium solani interactions Plant Physiol 1980 66 205 211 16661405 10.1104/pp.66.2.205 PMC440566

[B14] Plascencia-Jatomea M Viniegra G Olayo R Castillo-Ortega MM Shirai K Effect of chitosan and temperature on spore germination of Aspergillus niger Macromol Biosci 2003 3 582 586

[B15] Reddy MVB Arul J Ait-Barka E Angers P Richard C Castaigne F Effect of chitosan on growth and toxin production by Alternaria alternata f. sp. lycopersici Biocontrol Sci Techn 1998 8 33 43

[B16] Mellegard H Strand SP Christensen BE Granum PE Hardy SP Antibacterial activity of chemically defined chitosans: Influence of molecular weight, degree of acetylation and test organism Int J Food Microbiol 2011 148 48 54 21605923 10.1016/j.ijfoodmicro.2011.04.023

[B17] Stössel P Leuba JL Effect of chitosan, chitin and some aminosugars on growth of various soilborne phytopathogenic fungi J Phytopathol 1984 111 82 90

[B18] Liu H Du Y Wang X Sun L Chitosan kills bacteria through cell membrane damage Int J Food Microbiol 2004 95 147 155 15282127 10.1016/j.ijfoodmicro.2004.01.022

[B19] Liu XF Guan YL Yang DZ Li Z De Yao K Antibacterial action of chitosan and carboxymethylated chitosan J Appl Polym Sci 2001 79 1324 1335

[B20] Tikhonov V Stepnova E Babak V Yamskov I Palma-Guerrero J Jansson H Lopez-Llorca L Salinas J Gerasimenko D Avdienko I Varlamov V Bactericidal and antifungal activities of a low molecular weight chitosan and its N-/2(3)-(dodec-2-enyl)succinoyl/-derivatives Carbohyd Polym 2006 64 66 72

[B21] Helander IM Nurmiaho-Lassila EL Ahvenainen R Rhoades J Roller S Chitosan disrupts the barrier properties of the outer membrane of gram-negative bacteria Int J Food Microbiol 2001 71 235 244 11789941 10.1016/s0168-1605(01)00609-2

[B22] Zakrzewska A Boorsma A Brul S Hellingwerf KJ Klis FM Transcriptional response of Saccharomyces cerevisiae to the plasma membrane-perturbing compound chitosan Eukaryot Cell 2005 4 703 715 15821130 10.1128/EC.4.4.703-715.2005 PMC1087819

[B23] Zakrzewska A Boorsma A Delneri D Brul S Oliver SG Klis FM Cellular processes and pathways that protect Saccharomyces cerevisiae cells against the plasma membrane-perturbing compound chitosan Eukaryot Cell 2007 6 600 608 17259547 10.1128/EC.00355-06 PMC1865647

[B24] Ericson E Hoon S St Onge RP Giaever G Nislow C Exploring gene function and drug action using chemogenomic dosage assays Methods Enzymol 2010 470 233 255 20946813 10.1016/S0076-6879(10)70010-0

[B25] Hoon S Smith AM Wallace IM Suresh S Miranda M Fung E Proctor M Shokat KM Zhang C Davis RW An integrated platform of genomic assays reveals small-molecule bioactivities Nat Chem Biol 2008 4 498 506 18622389 10.1038/nchembio.100

[B26] Giaever G Flaherty P Kumm J Proctor M Nislow C Jaramillo DF Chu AM Jordan MI Arkin AP Davis RW Chemogenomic profiling: identifying the functional interactions of small molecules in yeast Proc Natl Acad Sci U S A 2004 101 793 798 14718668 10.1073/pnas.0307490100 PMC321760

[B27] Amor JC Horton JR Zhu X Wang Y Sullards C Ringe D Cheng X Kahn RA Structures of yeast ARF2 and ARL1: distinct roles for the N terminus in the structure and function of ARF family GTPases J Biol Chem 2001 276 42477 42484 11535602 10.1074/jbc.M106660200

[B28] Lee FJ Huang CF Yu WL Buu LM Lin CY Huang MC Moss J Vaughan M Characterization of an ADP-ribosylation factor-like 1 protein in Saccharomyces cerevisiae J Biol Chem 1997 272 30998 31005 9388248 10.1074/jbc.272.49.30998

[B29] Subramanian A Tamayo P Mootha VK Mukherjee S Ebert BL Gillette MA Paulovich A Pomeroy SL Golub TR Lander ES Mesirov JP Gene set enrichment analysis: a knowledge-based approach for interpreting genome-wide expression profiles Proc Natl Acad Sci U S A 2005 102 15545 15550 16199517 10.1073/pnas.0506580102 PMC1239896

[B30] Magtanong L Ho CH Barker SL Jiao W Baryshnikova A Bahr S Smith AM Heisler LE Choy JS Kuzmin E Dosage suppression genetic interaction networks enhance functional wiring diagrams of the cell Nat Biotechnol 2011 29 505 511 21572441 10.1038/nbt.1855 PMC7386433

[B31] Martin-Yken H Dagkessamanskaia A De Groot P Ram A Klis F Francois J Saccharomyces cerevisiae YCRO17c/CWH43 encodes a putative sensor/transporter protein upstream of the BCK2 branch of the PKC1-dependent cell wall integrity pathway Yeast 2001 18 827 840 11427965 10.1002/yea.731

[B32] Lee KS Irie K Gotoh Y Watanabe Y Araki H Nishida E Matsumoto K Levin DE A yeast mitogen-activated protein kinase homolog (Mpk1p) mediates signalling by protein kinase C Mol Cell Biol 1993 13 3067 3075 8386319 10.1128/mcb.13.5.3067-3075.1993 PMC359699

[B33] Flandez M Cosano IC Nombela C Martin H Molina M Reciprocal regulation between Slt2 MAPK and isoforms of Msg5 dual-specificity protein phosphatase modulates the yeast cell integrity pathway J Biol Chem 2004 279 11027 11034 14703512 10.1074/jbc.M306412200

[B34] Nordle AK Rios P Gaulton A Pulido R Attwood TK Tabernero L Functional assignment of MAPK phosphatase domains Proteins 2007 69 19 31 17596826 10.1002/prot.21477

[B35] Medina I Carbonell J Pulido L Madeira SC Goetz S Conesa A Tárraga J Pascual-Montano A Nogales-Cadenas R Santoyo J Babelomics: an integrative platform for the analysis of transcriptomics, proteomics and genomic data with advanced functional profiling Nucleic Acids Res 2010 38 W210 W213 20478823 10.1093/nar/gkq388 PMC2896184

[B36] Al-Shahrour F Carbonell J Minguez P Goetz S Conesa A Tárraga J Medina I Alloza E Montaner D Dopazo J Babelomics: advanced functional profiling of transcriptomics, proteomics and genomics experiments Nucleic Acids Res 2008 36 W341 W346 18515841 10.1093/nar/gkn318 PMC2447758

[B37] Thevissen K Terras FR Broekaert WF Permeabilization of fungal membranes by plant defensins inhibits fungal growth Appl Environ Microbiol 1999 65 5451 5458 10584003 10.1128/aem.65.12.5451-5458.1999 PMC91743

[B38] Berry DB Gasch AP Stress-activated genomic expression changes serve a preparative role for impending stress in yeast Mol Biol Cell 2008 19 4580 4587 18753408 10.1091/mbc.E07-07-0680 PMC2575158

[B39] Roberts CJ Nelson B Marton MJ Stoughton R Meyer MR Bennett HA He YD Dai H Walker WL Hughes TR Signaling and circuitry of multiple MAPK pathways revealed by a matrix of global gene expression profiles Science 2000 287 873 880 10657304 10.1126/science.287.5454.873

[B40] Ostrosky-Zeichner L Casadevall A Galgiani JN Odds FC Rex JH An insight into the antifungal pipeline: selected new molecules and beyond Nat Rev Drug Discov 2010 9 719 727 20725094 10.1038/nrd3074

[B41] Kong M Chen XG Xing K Park HJ Antimicrobial properties of chitosan and mode of action: A state of the art review Int J Food Microbiol 2010 144 51 63 20951455 10.1016/j.ijfoodmicro.2010.09.012

[B42] Xia W Liu P Zhang J Chen J Biological activities of chitosan and chitooligosaccharides Food Hydrocolloids 2011 25 170 179

[B43] Pierce SE Davis RW Nislow C Giaever G Genome-wide analysis of barcoded Saccharomyces cerevisiae gene-deletion mutants in pooled cultures Nat Protoc 2007 2 2958 2974 18007632 10.1038/nprot.2007.427

[B44] Munson AM Haydon DH Love SL Fell GL Palanivel VR Rosenwald AG Yeast ARL1 encodes a regulator of K + influx J Cell Sci 2004 117 2309 2320 15126631 10.1242/jcs.01050

[B45] Munro S The Arf-like GTPase Arl1 and its role in membrane traffic Biochem Soc Trans 2005 33 601 605 16042553 10.1042/BST0330601

[B46] Marešová L Sychrová H Genetic interactions among the Arl1 GTPase and intracellular Na(+)/H(+) antiporters in pH homeostasis and cation detoxification FEMS Yeast Res 2010 10 802 811 20659170 10.1111/j.1567-1364.2010.00661.x

[B47] Rosenwald AG Rhodes MA Van Valkenburgh H Palanivel V Chapman G Boman A Zhang CJ Kahn RA ARL1 and membrane traffic in Saccharomyces cerevisiae Yeast 2002 19 1039 1056 12210899 10.1002/yea.897

[B48] Bonangelino CJ Chavez EM Bonifacino JS Genomic screen for vacuolar protein sorting genes in Saccharomyces cerevisiae Mol Biol Cell 2002 13 2486 2501 12134085 10.1091/mbc.02-01-0005 PMC117329

[B49] Lomize AL Pogozheva ID Lomize MA Mosberg HI Positioning of proteins in membranes: a computational approach Protein Sci 2006 15 1318 1333 16731967 10.1110/ps.062126106 PMC2242528

[B50] Lomize MA Lomize AL Pogozheva ID Mosberg HI OPM: orientations of proteins in membranes database Bioinformatics 2006 22 623 625 16397007 10.1093/bioinformatics/btk023

[B51] Palma-Guerrero J Lopez-Jimenez JA Pérez-Berná AJ Huang I-C Jansson H-B Salinas J Villalaín J Read ND Lopez-Llorca LV Membrane fluidity determines sensitivity of filamentous fungi to chitosan Mol Microbiol 2010 75 1021 1032 20487294 10.1111/j.1365-2958.2009.07039.x

[B52] Opekarova M Malinsky J Tanner W Plants and fungi in the era of heterogeneous plasma membranes Plant Biol (Stuttg) 2010 12 Suppl 1 94 98 20712624 10.1111/j.1438-8677.2010.00356.x

[B53] Hardie DG Carling D The AMP-activated protein kinase–fuel gauge of the mammalian cell? Eur J Biochem 1997 246 259 273 9208914 10.1111/j.1432-1033.1997.00259.x

[B54] Palma-Guerrero J Huang I-C Jansson H-B Salinas J Lopez-Llorca LV Read ND Chitosan permeabilizes the plasma membrane and kills cells of Neurospora crassa in an energy dependent manner Fungal Genet Biol 2009 46 585 594 19389478 10.1016/j.fgb.2009.02.010

[B55] Palma-Guerrero J Jansson H-B Salinas J Lopez-Llorca LV Effect of chitosan on hyphal growth and spore germination of plant pathogenic and biocontrol fungi J Appl Microbiol 2008 104 541 553 17927761 10.1111/j.1365-2672.2007.03567.x

[B56] Gomez-Rivas L Escudero-Abarca BI Aguilar-Uscanga MG Hayward-Jones PM Mendoza P Ramirez M Selective antimicrobial action of chitosan against spoilage yeasts in mixed culture fermentations J Ind Microbiol Biotechnol 2004 31 16 22 14747932 10.1007/s10295-004-0112-2

[B57] Hillenmeyer ME Fung E Wildenhain J Pierce SE Hoon S Lee W Proctor M St Onge RP Tyers M Koller D The chemical genomic portrait of yeast: uncovering a phenotype for all genes Science 2008 320 362 365 18420932 10.1126/science.1150021 PMC2794835

[B58] Sheehan DJ Hitchcock CA Sibley CM Current and emerging azole antifungal agents Clin Microbiol Rev 1999 12 40 79 9880474 10.1128/cmr.12.1.40 PMC88906

[B59] Odds FC Brown AJ Gow NA Antifungal agents: mechanisms of action Trends Microbiol 2003 11 272 279 12823944 10.1016/s0966-842x(03)00117-3

[B60] Gruszecki WI Gagos M Herec M Kernen P Organization of antibiotic amphotericin B in model lipid membranes. A mini review Cell Mol Biol Lett 2003 8 161 170 12655370

[B61] Brajtburg J Powderly WG Kobayashi GS Medoff G Amphotericin B: current understanding of mechanisms of action Antimicrob Agents Chemother 1990 34 183 188 2183713 10.1128/aac.34.2.183 PMC171553

[B62] Bottema CK Parks LW Delta14-sterol reductase in Saccharomyces cerevisiae Biochim Biophys Acta 1978 531 301 307 32908 10.1016/0005-2760(78)90212-6

[B63] Burke D Methods in yeast genetics: a Cold Spring Harbor Laboratory course manual 2000 Cold Spring Harbor Laboratory Press, Plainview, N.Y.

[B64] Gietz RD Schiestl RH Large-scale high-efficiency yeast transformation using the LiAc/SS carrier DNA/PEG method Nat Protoc 2007 2 38 41 17401336 10.1038/nprot.2007.15

[B65] Chomczynski P Sacchi N Single-step method of RNA isolation by acid guanidinium thiocyanate-phenol-chloroform extraction Anal Biochem 1987 162 156 159 2440339 10.1006/abio.1987.9999

[B66] Carvalho BS Irizarry RA A framework for oligonucleotide microarray preprocessing Bioinformatics 2010 26 2363 2367 20688976 10.1093/bioinformatics/btq431 PMC2944196

[B67] Smyth G Thorne N Wettenhall J Limma: linear models for microarray data user’s guide Software manual available from http://www bioconductor org 2003

[B68] Edgar R Domrachev M Lash AE Gene Expression Omnibus: NCBI gene expression and hybridization array data repository Nucleic Acids Res 2002 30 207 210 11752295 10.1093/nar/30.1.207 PMC99122

[B69] Eisen MB Spellman PT Brown PO Botstein D Cluster analysis and display of genome-wide expression patterns Proc Natl Acad Sci U S A 1998 95 14863 14868 9843981 10.1073/pnas.95.25.14863 PMC24541

[B70] de Hoon MJ Imoto S Nolan J Miyano S Open source clustering software Bioinformatics 2004 20 1453 1454 14871861 10.1093/bioinformatics/bth078

[B71] Saldanha AJ Java Treeview–extensible visualization of microarray data Bioinformatics 2004 20 3246 3248 15180930 10.1093/bioinformatics/bth349

[B72] Benschop JJ Brabers N van Leenen D Bakker LV van Deutekom HW van Berkum NL Apweiler E Lijnzaad P Holstege FC Kemmeren P A consensus of core protein complex compositions for Saccharomyces cerevisiae Mol Cell 2010 38 916 928 20620961 10.1016/j.molcel.2010.06.002

[B73] Merico D Isserlin R Stueker O Emili A Bader GD Enrichment map: a network-based method for gene-set enrichment visualization and interpretation PLoS One 2010 5 e13984 21085593 10.1371/journal.pone.0013984 PMC2981572

[B74] Smoot ME Ono K Ruscheinski J Wang PL Ideker T Cytoscape 2.8: new features for data integration and network visualization Bioinformatics 2011 27 431 432 21149340 10.1093/bioinformatics/btq675 PMC3031041

[B75] Gasch AP Spellman PT Kao CM Carmel-Harel O Eisen MB Storz G Botstein D Brown PO Genomic expression programs in the response of yeast cells to environmental changes Mol Biol Cell 2000 11 4241 4257 11102521 10.1091/mbc.11.12.4241 PMC15070

[B76] Rozen S Skaletsky H Primer3 on the WWW for general users and for biologist programmers Methods Mol Biol 2000 132 365 386 10547847 10.1385/1-59259-192-2:365

