# Supplementary material for: Identification of yeast genes that confer resistance to chitosan oligosaccharide (COS) using chemogenomics
Source: BMC Genomics. 2012 Jun 22;13:267. doi: 10.1186/1471-2164-13-267 (PMC3505485; doi:10.1186/1471-2164-13-267)
Supplement: Additional file 1 — Figures S1 – S11 and their corresponding figure legends. [file 1471-2164-13-267-S1.pdf]

Additional file 1 – Supplementary figures S1 - S11 and their corresponding figure legends (PDF, This file can be viewed with: Adobe Acrobat Reader).

Figure S1. Chitosan oligosaccharide (COS-5.44) has higher antifungal activity than higher molecular weight chitosan (T8s). Chitosan oligosaccharide (5.44 kDa) and chitosan (70KDa) were screened in wild type yeast (BY4743) in 0.5X YPD pH 5. Five concentrations of COS-5.44 (91.1 – 112.5 μg/ml) and 4 of chitosan (125 – 162.5 μg/ml) were tested. Optical density readings were taken every 15 minutes over 20 hrs using a Tecan Genios reader (see Methods).

Figure S2. Multicopy suppression profile assay (MSP) of yeast growing in 0.5X YPD pH 5 with 112.5 μg/ml of chitosan oligosaccharide (COS-5.44). The scatter plot shows the correlation between the two biological replicates of the multicopy suppression profile assay (MSP). Sixty-eight genes are potential suppressors to COS-5.44 sensitivity. Potential suppressors identified by replicate 1 are shown in red, replicate 2, blue and in both replicates, purple. The twenty-one strains confirmed as suppressors are indicated by their gene name. The *ARL1* suppressor strain was also found in the HIP-HOP assay as a sensitive deletion strain (highlighted).

Figure S3. Enriched biological processes from transcript changes in COS and Chitosan treated cells. The GSEA enrichment map displays a comparison of enriched terms from the transcriptional profile of COS treated wild type cells from our study with transcript data from chitosan treated cells from Zakrzewska et al. (2005). Enrichments were mapped to the inner node area and to the node border, respectively. A node represents a biological process significantly enriched (FDR ≤ 0.001, see Methods). The node size correlates to the number of genes annotated to that functional category. Red and blue node border colours indicate the biological processes that are enriched in the up- and down-regulated gene transcripts respectively. The thickness of the edge correlates to the degree of gene overlap between the two connected categories. If the overlap coefficient is less than 0.5 edges are not shown (see Methods). Cluster relationship is shown by edge colour, where blue edges represent "gene overlap/relationship among biological processes" in our study and green in the Zakrzewska et al. (2005) data set. Clustering of terms is based on degree of overlap of genes among the categories.

Figure S4. Hierarchical clustering of the expression profiles of COS-5.44 resistant yeast overexpressing strains compared with the wild type in the absence of COS-5.44. One hundred and eighty four genes showed a significant change in expression (P-value ≤ 0.05 and log2 fold change ≥ 1 or < -1) in at least one of the overexpression strains compared with the wild type basal expression, clustering was based in similarity (see Methods). A) Significantly up-regulated genes in *Arl1* and *Rba50* overexpressing strains. B) *RBA50* gene is up-regulated in the corresponding overexpressing strain. C) Cluster of down-regulated genes among the 5 overexpressing strains, except that *MSG5* is up-regulated in the corresponding overexpressing strain. D) Cluster of up-regulated genes among the 5 overexpressing strains.

Figure S5. Biological processes associated with differentially expressed genes in an *Arl1* overexpression strain when exposed to chitosan oligosaccharide (COS). A node represents a biological process significantly enriched (FDR $\leq$ 0.1, see Methods). Boxes on the side show summary of the main biological process found in a cluster. The node size correlates to the number of genes annotated to that functional category. Red and blue node border colours indicate the biological processes that are enriched in the up- and down-regulated gene transcripts respectively. The width of the edge correlates to the degree of gene overlap between the two connected categories. If the overlap coefficient is less than 0.5, edges are not shown (see Methods). Cluster of terms is shown by node color, where clustering is based on degree of overlap of genes among the categories. Bar plots show log fold change of the genes that contributed to the functional enrichment of the cluster, with the border color surrounding the plots correlating with the nodes in the cluster. The top 10 genes in each cluster category are shown in each plot.

Figure S6. Biological process associated with differentially expressed genes on *Bck2* overexpression strain when exposed to chitosan oligosaccharide (COS). For details in annotation refer to Figure S5.

Figure S7. Biological process associated with differentially expressed genes on *Erg24* overexpression strain when exposed to chitosan oligosaccharide (COS). For details in annotation refer to Figure S5.

Figure S8. Biological process associated with differentially expressed genes on *Msg5* overexpression strain when exposed to chitosan oligosaccharide (COS). For details in annotation refer to Figure S5.

Figure S9. Biological process associated with differentially expressed genes on *Rba50* overexpression strain when exposed to chitosan oligosaccharide (COS). For details in annotation refer to Figure S5.

Figure S10. Quantitative RT-PCR verification of the microarray data for 4 up-regulated (*VBA5*, *MUC1*, *YJU2* and *FIG2*) and 5 down-regulated genes (*CMD1*, *COX5b*, *UBI4*, *RCR1* and *HSP30*) yields similar results. Two biological replicates from the wild type (vector control BY4743) and the *Arl1* overexpressing strain that were either untreated or exposed to COS-5.44 for 60 min were used for qRT-PCR. Expressed as log2 fold change as relative expression to the wild type samples.

Figure S11. Effect of yeast environmental stress response agents on yeast WT (BY4743) grown in 0.5X YPD pH 5, amended with chitosan oligosaccharide (COS-4.55). Wild type cells do not acquire resistance to COS-4.55 treatment after exposure to sub lethal doses of thermal (B), salt (C), osmotic (D) and oxidative (E-F) stresses. A) Mock Control (normal growth conditions).

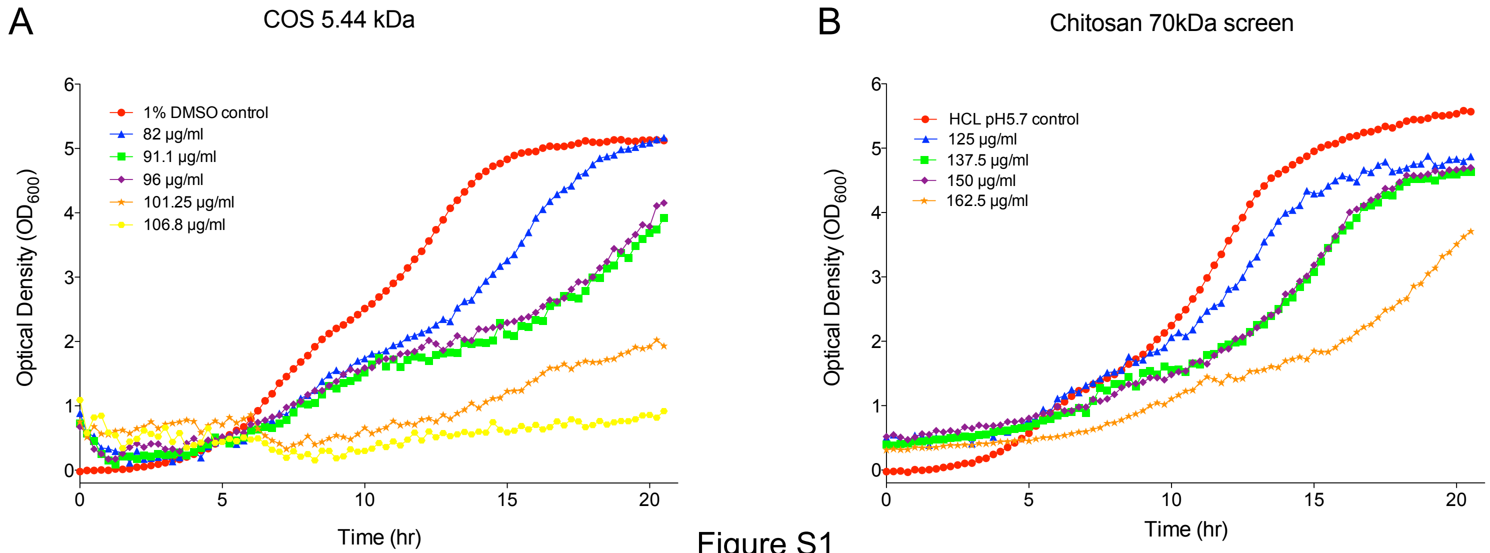
A
COS 5.44 kDa
1% DMSO control
82 µg/ml
91.1 µg/ml
96 µg/ml
101.25 µg/ml
106.8 µg/ml
Optical Density (OD_{600})
Time (hr)
B
Chitosan 70kDa screen
HCL pH5.7 control
125 µg/ml
137.5 µg/ml
150 µg/ml
162.5 µg/ml
Optical Density (OD_{600})
Time (hr)

Figure S1

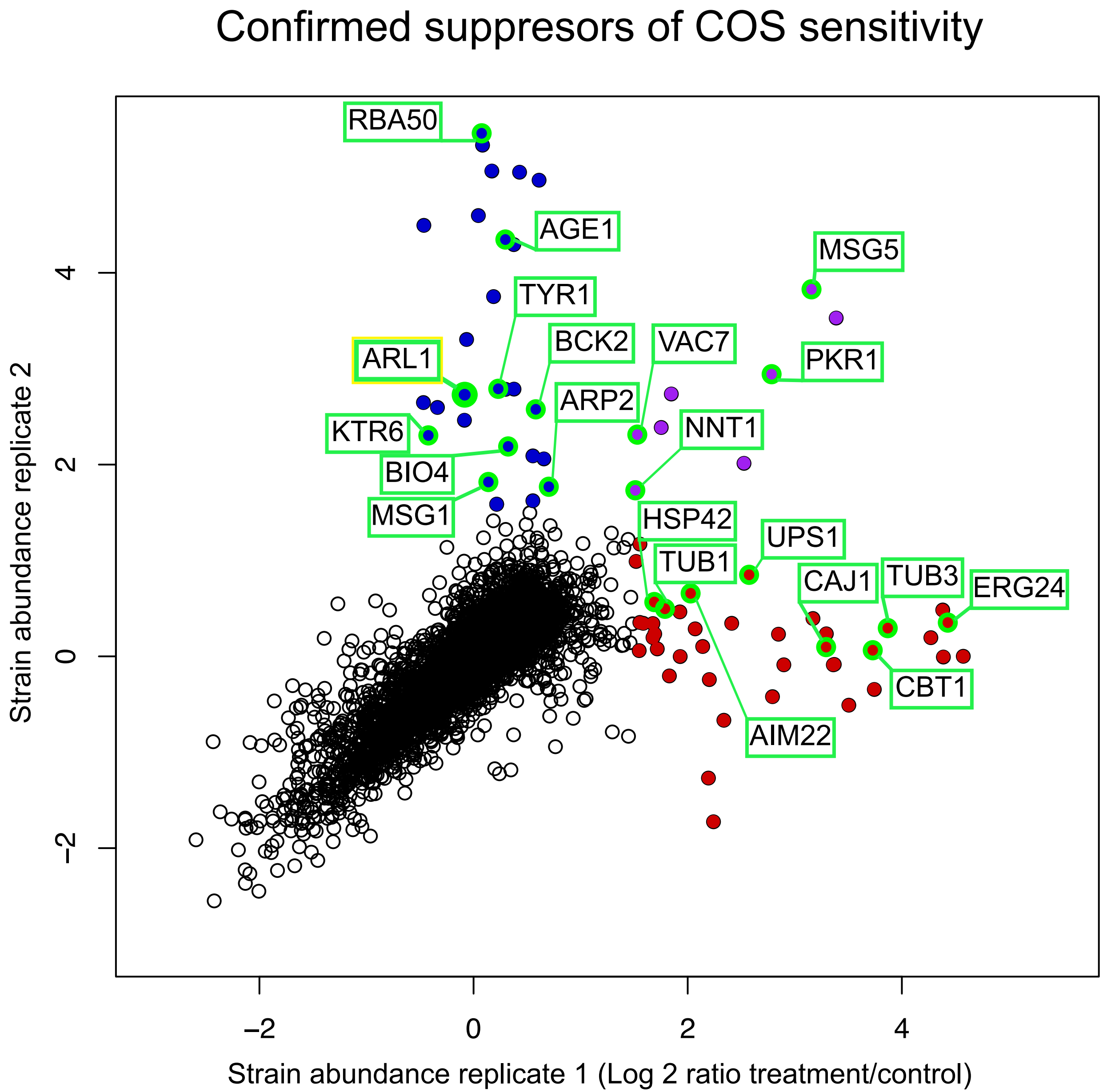
Confirmed suppresors of COS sensitivity
Strain abundance replicate 2
Strain abundance replicate 1 (Log 2 ratio treatment/control)
4
2
0
−2
−2
0
2
4
RBA50
AGE1
MSG5
TYR1
BCK2
VAC7
ARL1
PKR1
ARP2
KTR6
NNT1
BIO4
MSG1
HSP42
UPS1
TUB1
CAJ1
TUB3
ERG24
CBT1
AIM22
Figure S2

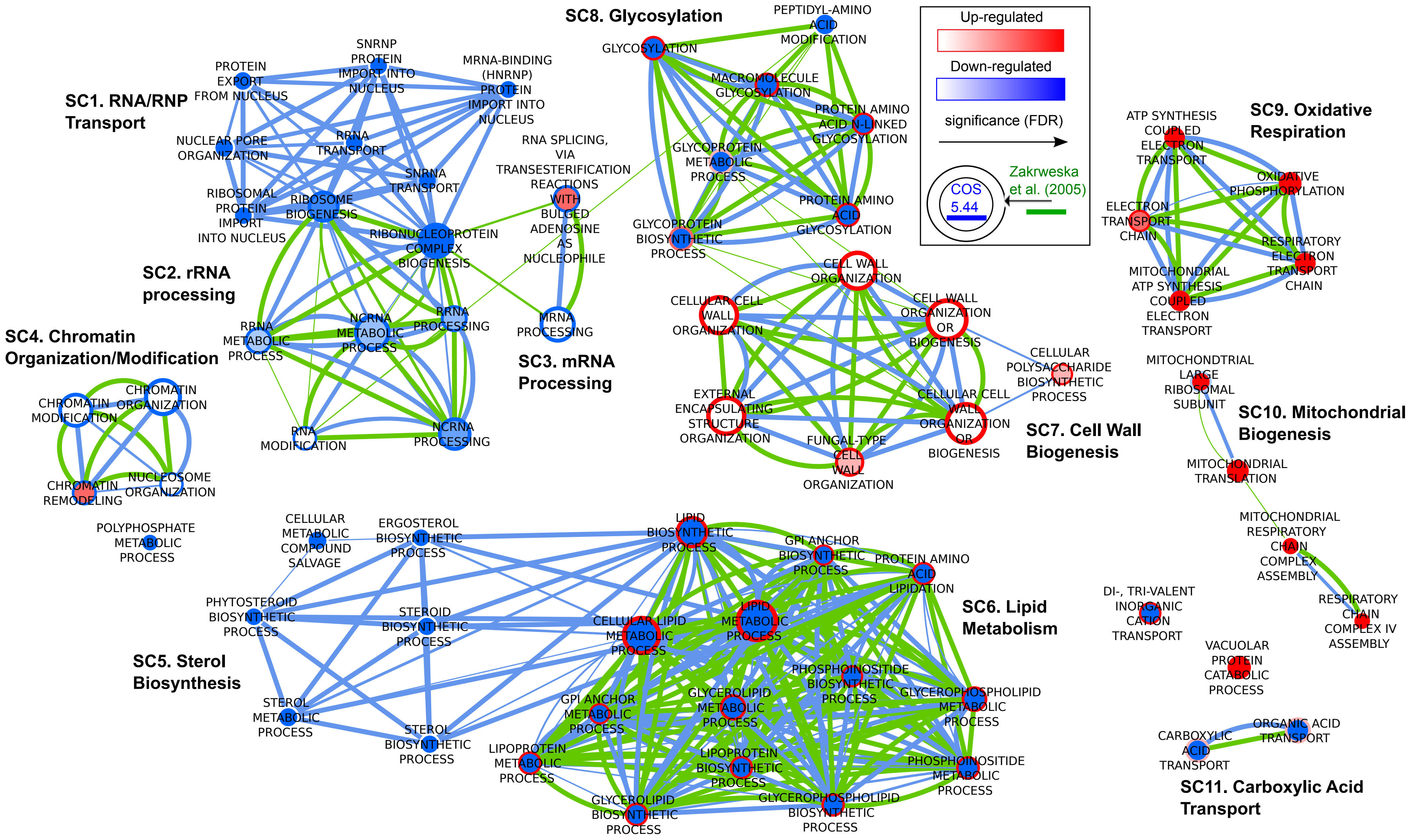

Up-regulated
Down-regulated
significance (FDR)
COS
5.44
Zakrweska et al. (2005)
SC1. RNA/RNP Transport
PROTEIN EXPORT FROM NUCLEUS
SNRNP PROTEIN IMPORT INTO NUCLEUS
MRNA-BINDING (HNRNP) PROTEIN IMPORT INTO NUCLEUS
NUCLEAR PORE ORGANIZATION
RRNA TRANSPORT
SNRNA TRANSPORT
RIBOSOMAL PROTEIN IMPORT INTO NUCLEUS
RIBOSOME BIOGENESIS
RIBONUCLEOPROTEIN COMPLEX BIOGENESIS
SC2. rRNA processing
RRNA METABOLIC PROCESS
NCRNA METABOLIC PROCESS
RRNA PROCESSING
RNA MODIFICATION
NCRNA PROCESSING
RNA SPLICING, VIA TRANSESTERIFICATION REACTIONS WITH BULGED ADENOSINE AS NUCLEOPHILE
MRNA PROCESSING
SC3. mRNA Processing
SC4. Chromatin Organization/Modification
CHROMATIN MODIFICATION
CHROMATIN ORGANIZATION
CHROMATIN REMODELING
NUCLEOSOME ORGANIZATION
POLYPHOSPHATE METABOLIC PROCESS
SC8. Glycosylation
GLYCOSYLATION
PEPTIDYL-AMINO ACID MODIFICATION
MACROMOLECULE GLYCOSYLATION
PROTEIN AMINO ACID N-LINKED GLYCOSYLATION
GLYCOPROTEIN METABOLIC PROCESS
PROTEIN AMINO ACID GLYCOSYLATION
GLYCOPROTEIN BIOSYNTHETIC PROCESS
CELL WALL ORGANIZATION
CELLULAR CELL WALL ORGANIZATION
CELL WALL ORGANIZATION OR BIOGENESIS
CELLULAR POLYSACCHARIDE BIOSYNTHETIC PROCESS
EXTERNAL ENCAPSULATING STRUCTURE ORGANIZATION
CELLULAR CELL WALL ORGANIZATION OR BIOGENESIS
FUNGAL-TYPE CELL WALL ORGANIZATION
SC7. Cell Wall Biogenesis
SC9. Oxidative Respiration
ATP SYNTHESIS COUPLED ELECTRON TRANSPORT
OXIDATIVE PHOSPHORYLATION
ELECTRON TRANSPORT CHAIN
RESPIRATORY ELECTRON TRANSPORT CHAIN
MITOCHONDRIAL ATP SYNTHESIS COUPLED ELECTRON TRANSPORT
MITOCHONDTRIAL LARGE RIBOSOMAL SUBUNIT
SC10. Mitochondrial Biogenesis
MITOCHONDRIAL TRANSLATION
MITOCHONDRIAL RESPIRATORY CHAIN COMPLEX ASSEMBLY
RESPIRATORY CHAIN COMPLEX IV ASSEMBLY
DI-, TRI-VALENT INORGANIC CATION TRANSPORT
VACUOLAR PROTEIN CATABOLIC PROCESS
CARBOXYLIC ACID TRANSPORT
ORGANIC ACID TRANSPORT
SC11. Carboxylic Acid Transport
CELLULAR METABOLIC COMPOUND SALVAGE
ERGOSTEROL BIOSYNTHETIC PROCESS
LIPID BIOSYNTHETIC PROCESS
GPI ANCHOR BIOSYNTHETIC PROCESS
PROTEIN AMINO ACID LIPIDATION
SC6. Lipid Metabolism
PHYTOSTEROID BIOSYNTHETIC PROCESS
STEROID BIOSYNTHETIC PROCESS
CELLULAR LIPID METABOLIC PROCESS
LIPID METABOLIC PROCESS
SC5. Sterol Biosynthesis
PHOSPHOINOSITIDE BIOSYNTHETIC PROCESS
GLYCEROLIPID METABOLIC PROCESS
GLYCEROPHOSPHOLIPID METABOLIC PROCESS
GPI ANCHOR METABOLIC PROCESS
STEROL METABOLIC PROCESS
STEROL BIOSYNTHETIC PROCESS
LIPOPROTEIN METABOLIC PROCESS
LIPOPROTEIN BIOSYNTHETIC PROCESS
PHOSPHOINOSITIDE METABOLIC PROCESS
GLYCEROLIPID BIOSYNTHETIC PROCESS
GLYCEROPHOSPHOLIPID BIOSYNTHETIC PROCESS

Figure S3

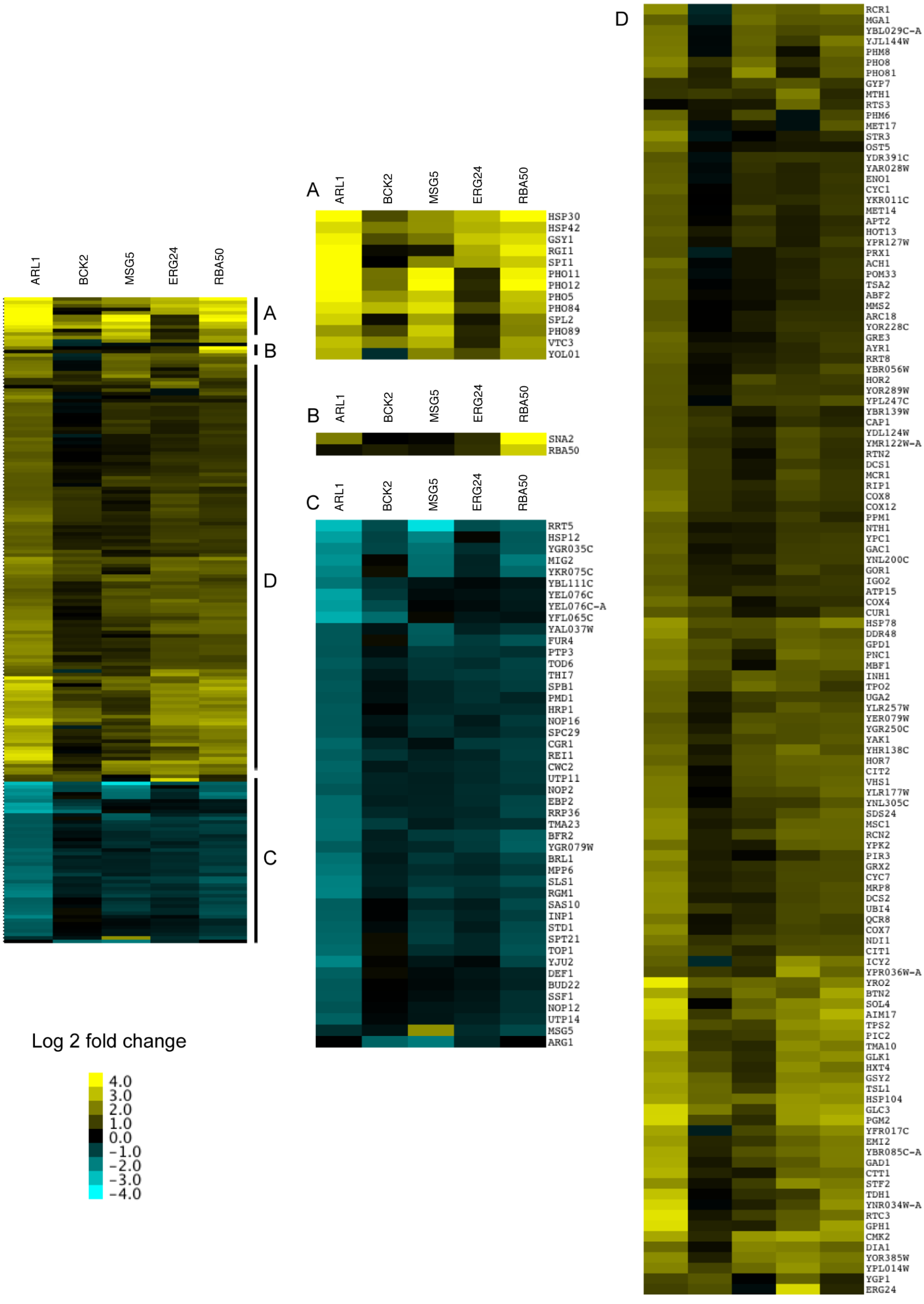

ARL1
BCK2
MSG5
ERG24
RBA50
A
B
C
D
Log 2 fold change
4.0
3.0
2.0
1.0
0.0
-1.0
-2.0
-3.0
-4.0
A
HSP30
HSP42
GSY1
RGI1
SPI1
PHO11
PHO12
PHO5
PHO84
SPL2
PHO89
VTC3
YOL01
B
SNA2
RBA50
C
RRT5
HSP12
YGR035C
MIG2
YKR075C
YBL111C
YEL076C
YEL076C-A
YFL065C
YAL037W
FUR4
PTP3
TOD6
THI7
SPB1
PMD1
HRP1
NOP16
SPC29
CGR1
REI1
CWC2
UTP11
NOP2
EBP2
RRP36
TMA23
BFR2
YGR079W
BRL1
MPP6
SLS1
RGM1
SAS10
INP1
STD1
SPT21
TOP1
YJU2
DEF1
BUD22
SSF1
NOP12
UTP14
MSG5
ARG1
D
RCR1
MGA1
YBL029C-A
YJL144W
PHM8
PHO8
PHO81
GYP7
MTH1
RTS3
PHM6
MET17
STR3
OST5
YDR391C
YAR028W
ENO1
CYC1
YKR011C
MET14
APT2
HOT13
YPR127W
PRX1
ACH1
POM33
TSA2
ABF2
MMS2
ARC18
YOR228C
GRE3
AYR1
RRT8
YBR056W
HOR2
YOR289W
YPL247C
YBR139W
CAP1
YDL124W
YMR122W-A
RTN2
DCS1
MCR1
RIP1
COX8
COX12
PPM1
NTH1
YPC1
GAC1
YNL200C
GOR1
IGO2
ATP15
COX4
CUR1
HSP78
DDR48
GPD1
PNC1
MBF1
INH1
TPO2
UGA2
YLR257W
YER079W
YGR250C
YAK1
YHR138C
HOR7
CIT2
VHS1
YLR177W
YNL305C
SDS24
MSC1
RCN2
YPK2
PIR3
GRX2
CYC7
MRP8
DCS2
UBI4
QCR8
COX7
NDI1
CIT1
ICY2
YPR036W-A
YRO2
BTN2
SOL4
AIM17
TPS2
PIC2
TMA10
GLK1
HXT4
GSY2
TSL1
HSP104
GLC3
PGM2
YFR017C
EMI2
YBR085C-A
GAD1
CTT1
STF2
TDH1
YNR034W-A
RTC3
GPH1
CMK2
DIA1
YOR385W
YPL014W
YGP1
ERG24

Figure S4

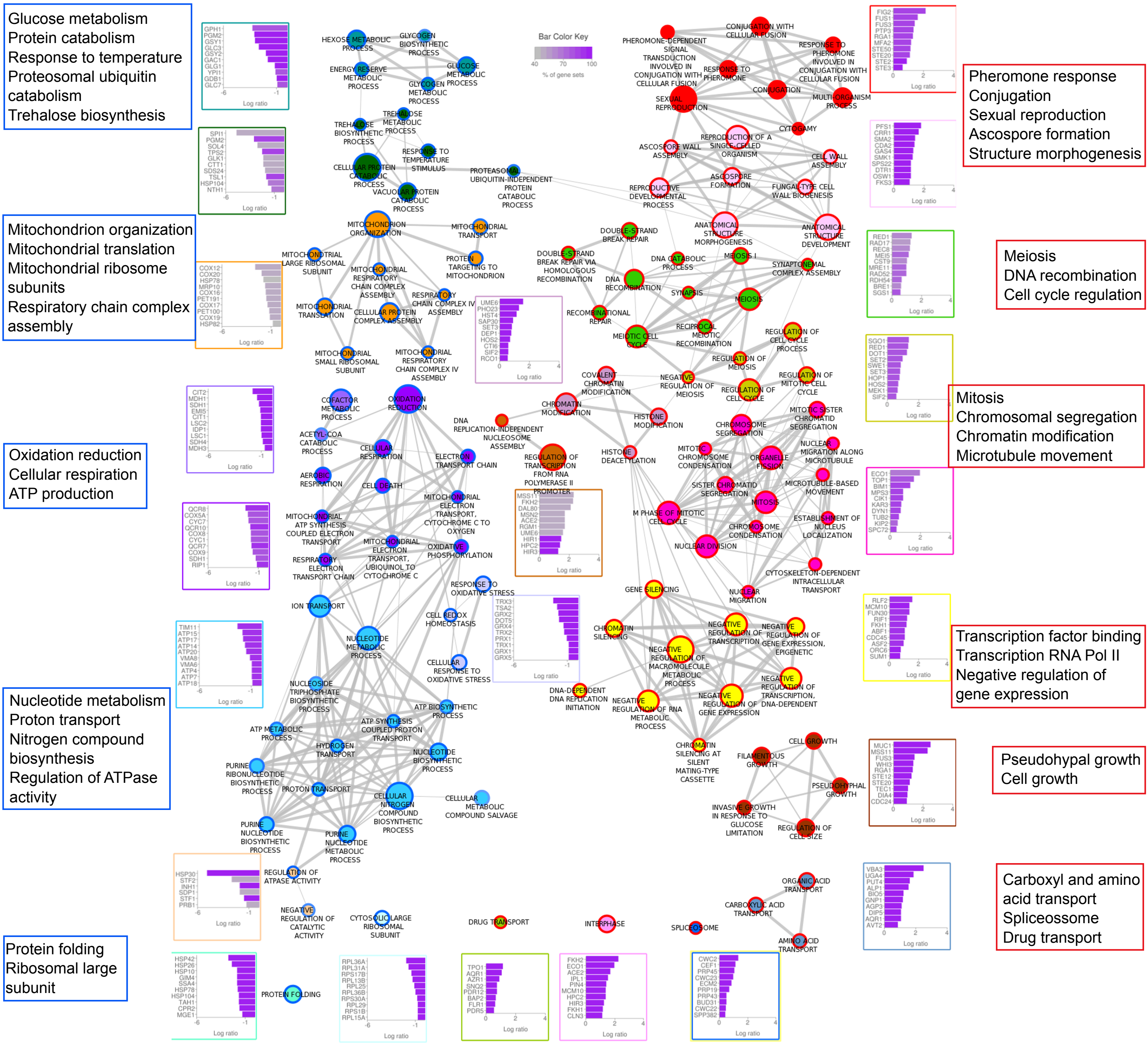
Glucose metabolism
Protein catabolism
Response to temperature
Proteosomal ubiquitin catabolism
Trehalose biosynthesis
Mitochondrion organization
Mitochondrial translation
Mitochondrial ribosome subunits
Respiratory chain complex assembly
Oxidation reduction
Cellular respiration
ATP production
Nucleotide metabolism
Proton transport
Nitrogen compound biosynthesis
Regulation of ATPase activity
Protein folding
Ribosomal large subunit
Pheromone response
Conjugation
Sexual reproduction
Ascospore formation
Structure morphogenesis
Meiosis
DNA recombination
Cell cycle regulation
Mitosis
Chromosomal segregation
Chromatin modification
Microtubule movement
Transcription factor binding
Transcription RNA Pol II
Negative regulation of gene expression
Pseudohypal growth
Cell growth
Carboxyl and amino acid transport
Spliceosome
Drug transport
Bar Color Key
% of gene sets
Log ratio

Figure S5

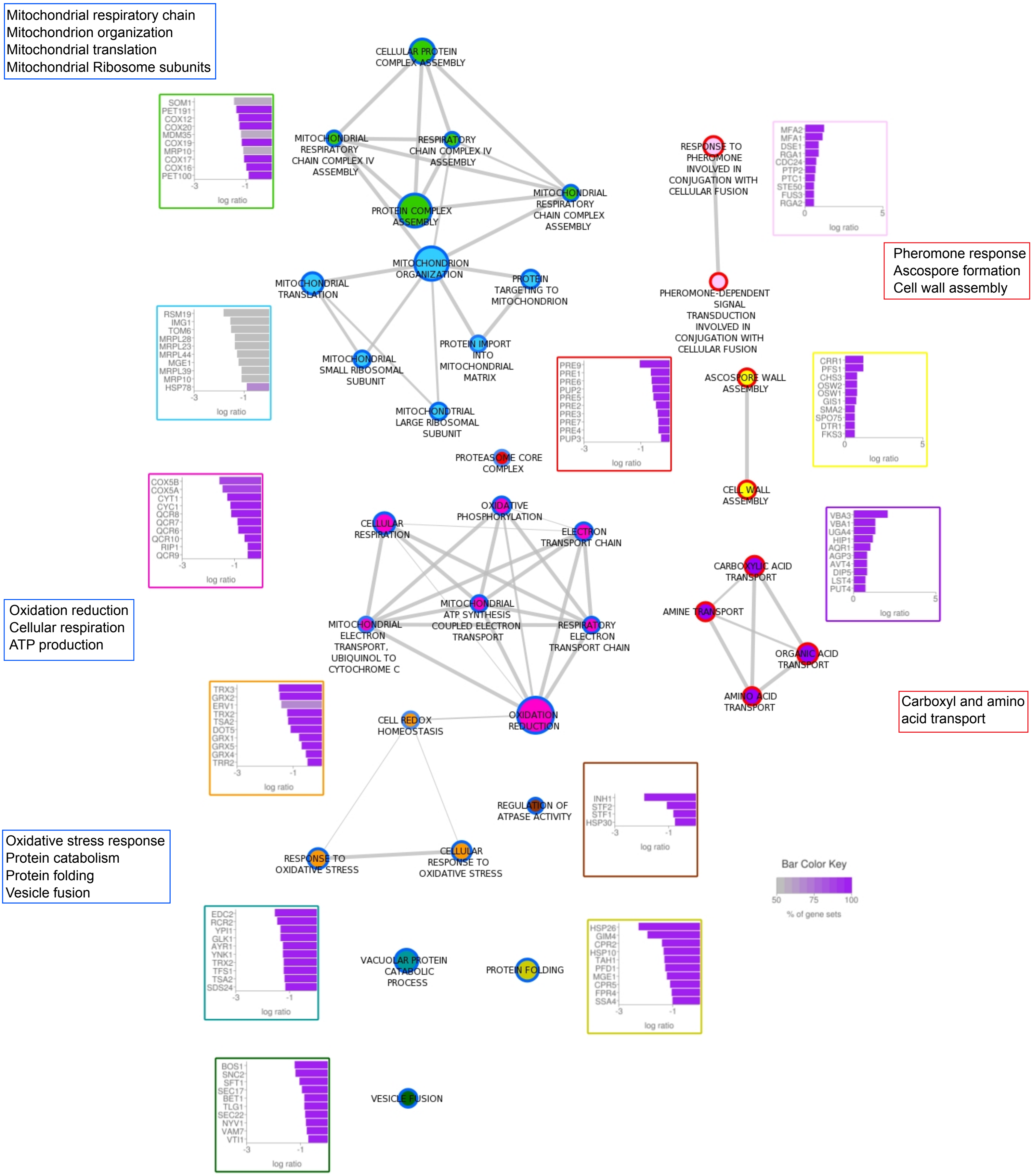

Mitochondrial respiratory chain
Mitochondrion organization
Mitochondrial translation
Mitochondrial Ribosome subunits
CELLULAR PROTEIN COMPLEX ASSEMBLY
MITOCHONDRIAL RESPIRATORY CHAIN COMPLEX IV ASSEMBLY
RESPIRATORY CHAIN COMPLEX IV ASSEMBLY
PROTEIN COMPLEX ASSEMBLY
MITOCHONDRIAL RESPIRATORY CHAIN COMPLEX ASSEMBLY
MITOCHONDRION ORGANIZATION
MITOCHONDRIAL TRANSLATION
PROTEIN TARGETING TO MITOCHONDRION
PROTEIN IMPORT INTO MITOCHONDRIAL MATRIX
MITOCHONDRIAL SMALL RIBOSOMAL SUBUNIT
MITOCHONDRIAL LARGE RIBOSOMAL SUBUNIT
PROTEASOME CORE COMPLEX
SOM1 PET191 COX12 COX20 MDM35 COX19 MRP10 COX17 COX16 PET100
RSM19 IMG1 TOM6 MRPL28 MRPL23 MRPL44 MGE1 MRPL39 MRP10 HSP78
PRE9 PRE1 PRE6 PUP2 PRE5 PRE2 PRE3 PRE7 PRE4 PUP3
log ratio
RESPONSE TO PHEROMONE INVOLVED IN CONJUGATION WITH CELLULAR FUSION
PHEROMONE-DEPENDENT SIGNAL TRANSDUCTION INVOLVED IN CONJUGATION WITH CELLULAR FUSION
MFA2 MFA1 DSE1 RGA1 CDC24 PTP2 PTC1 STE50 FUS3 RGA2
Pheromone response
Ascospore formation
Cell wall assembly
ASCOSPORE WALL ASSEMBLY
CELL WALL ASSEMBLY
CRR1 PFS1 CHS3 OSW2 OSW1 GIS1 SMA2 SPO75 DTR1 FKS3
COX5B COX5A CYT1 CYC1 QCR8 QCR7 QCR6 QCR10 RIP1 QCR9
OXIDATIVE PHOSPHORYLATION
CELLULAR RESPIRATION
ELECTRON TRANSPORT CHAIN
MITOCHONDRIAL ATP SYNTHESIS COUPLED ELECTRON TRANSPORT
MITOCHONDRIAL ELECTRON TRANSPORT, UBIQUINOL TO CYTOCHROME C
RESPIRATORY ELECTRON TRANSPORT CHAIN
OXIDATION REDUCTION
CELL REDOX HOMEOSTASIS
Oxidation reduction
Cellular respiration
ATP production
CARBOXYLIC ACID TRANSPORT
AMINE TRANSPORT
ORGANIC ACID TRANSPORT
AMINO ACID TRANSPORT
VBA3 VBA1 UGA4 HIP1 AQR1 AGP3 AVT4 DIP5 LST4 PUT4
Carboxyl and amino acid transport
TRX3 GRX2 ERV1 TRX2 TSA2 DOT5 GRX1 GRX5 GRX4 TRR2
REGULATION OF ATPASE ACTIVITY
INH1 STF2 STF1 HSP30
RESPONSE TO OXIDATIVE STRESS
CELLULAR RESPONSE TO OXIDATIVE STRESS
Oxidative stress response
Protein catabolism
Protein folding
Vesicle fusion
Bar Color Key
50 75 100
% of gene sets
EDC2 RCR2 YPI1 GLK1 AYR1 YNK1 TRX2 TFS1 TSA2 SDS24
VACUOLAR PROTEIN CATABOLIC PROCESS
PROTEIN FOLDING
HSP26 GIM4 CPR2 HSP10 TAH1 PFD1 MGE1 CPR5 FPR4 SSA4
BOS1 SNC2 SFT1 SEC17 BET1 TLG1 SEC22 NYV1 VAM7 VTI1
VESICLE FUSION

Figure S6

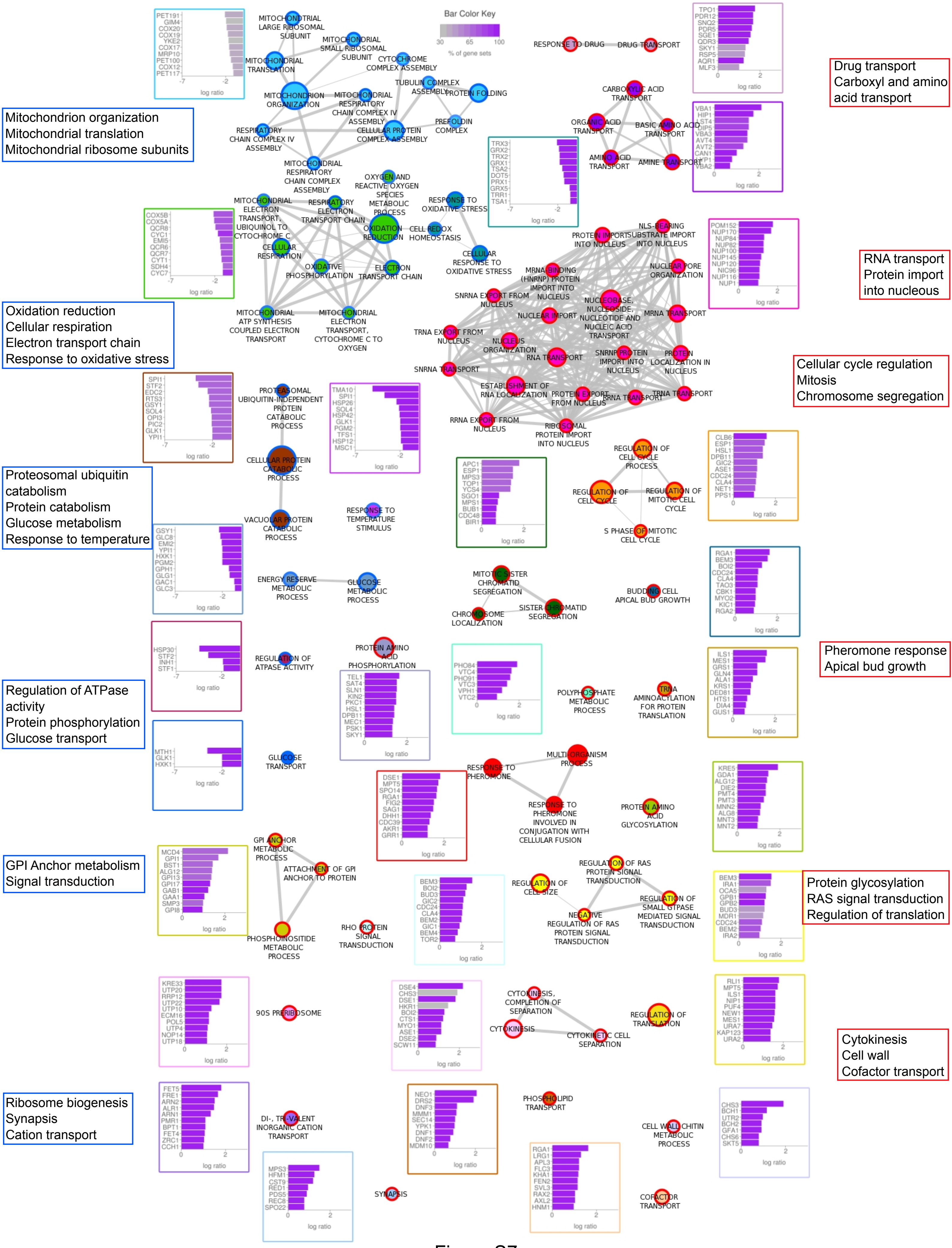

Bar Color Key
% of gene sets
log ratio
Mitochondrion organization
Mitochondrial translation
Mitochondrial ribosome subunits
Drug transport
Carboxyl and amino acid transport
Oxidation reduction
Cellular respiration
Electron transport chain
Response to oxidative stress
RNA transport
Protein import into nucleous
Cellular cycle regulation
Mitosis
Chromosome segregation
Proteosomal ubiquitin catabolism
Protein catabolism
Glucose metabolism
Response to temperature
Pheromone response
Apical bud growth
Regulation of ATPase activity
Protein phosphorylation
Glucose transport
GPI Anchor metabolism
Signal transduction
Protein glycosylation
RAS signal transduction
Regulation of translation
Cytokinesis
Cell wall
Cofactor transport
Ribosome biogenesis
Synapsis
Cation transport
MITOCHONDRIAL LARGE RIBOSOMAL SUBUNIT
MITOCHONDRIAL SMALL RIBOSOMAL SUBUNIT
MITOCHONDRIAL TRANSLATION
CYTOCHROME COMPLEX ASSEMBLY
TUBULIN COMPLEX ASSEMBLY
PROTEIN FOLDING
MITOCHONDRION ORGANIZATION
MITOCHONDRIAL RESPIRATORY CHAIN COMPLEX IV ASSEMBLY
PREFOLDIN COMPLEX
RESPIRATORY CHAIN COMPLEX IV ASSEMBLY
CELLULAR PROTEIN COMPLEX ASSEMBLY
RESPONSE TO DRUG
DRUG TRANSPORT
CARBOXYLIC ACID TRANSPORT
ORGANIC ACID TRANSPORT
BASIC AMINO ACID TRANSPORT
AMINO ACID TRANSPORT
AMINE TRANSPORT
MITOCHONDRIAL RESPIRATORY CHAIN COMPLEX ASSEMBLY
OXYGEN AND REACTIVE OXYGEN SPECIES METABOLIC PROCESS
RESPONSE TO OXIDATIVE STRESS
MITOCHONDRIAL ELECTRON TRANSPORT, UBIQUINOL TO CYTOCHROME C
RESPIRATORY ELECTRON TRANSPORT CHAIN
OXIDATION REDUCTION
CELL REDOX HOMEOSTASIS
CELLULAR RESPIRATION
CELLULAR RESPONSE TO OXIDATIVE STRESS
OXIDATIVE PHOSPHORYLATION
ELECTRON TRANSPORT CHAIN
MITOCHONDRIAL ATP SYNTHESIS COUPLED ELECTRON TRANSPORT
MITOCHONDRIAL ELECTRON TRANSPORT, CYTOCHROME C TO OXYGEN
PROTEIN IMPORT INTO NUCLEUS
NLS-BEARING SUBSTRATE IMPORT INTO NUCLEUS
MRNA BINDING (HNRNP) PROTEIN IMPORT INTO NUCLEUS
NUCLEAR PORE ORGANIZATION
SNRNA EXPORT FROM NUCLEUS
NUCLEOBASE, NUCLEOSIDE, NUCLEOTIDE AND NUCLEIC ACID TRANSPORT
NUCLEAR IMPORT
MRNA TRANSPORT
TRNA EXPORT FROM NUCLEUS
NUCLEUS ORGANIZATION
RNA TRANSPORT
SNRNP PROTEIN IMPORT INTO NUCLEUS
PROTEIN LOCALIZATION IN NUCLEUS
SNRNA TRANSPORT
ESTABLISHMENT OF RNA LOCALIZATION
PROTEIN EXPORT FROM NUCLEUS
TRNA TRANSPORT
RRNA EXPORT FROM NUCLEUS
RIBOSOMAL PROTEIN IMPORT INTO NUCLEUS
PROTEASOMAL UBIQUITIN-INDEPENDENT PROTEIN CATABOLIC PROCESS
CELLULAR PROTEIN CATABOLIC PROCESS
VACUOLAR PROTEIN CATABOLIC PROCESS
RESPONSE TO TEMPERATURE STIMULUS
REGULATION OF CELL CYCLE PROCESS
REGULATION OF CELL CYCLE
REGULATION OF MITOTIC CELL CYCLE
S PHASE OF MITOTIC CELL CYCLE
ENERGY RESERVE METABOLIC PROCESS
GLUCOSE METABOLIC PROCESS
MITOTIC SISTER CHROMATID SEGREGATION
CHROMOSOME LOCALIZATION
SISTER CHROMATID SEGREGATION
BUDDING CELL APICAL BUD GROWTH
REGULATION OF ATPASE ACTIVITY
PROTEIN AMINO ACID PHOSPHORYLATION
POLYPHOSPHATE METABOLIC PROCESS
TRNA AMINOACYLATION FOR PROTEIN TRANSLATION
GLUCOSE TRANSPORT
RESPONSE TO PHEROMONE
MULTI-ORGANISM PROCESS
RESPONSE TO PHEROMONE INVOLVED IN CONJUGATION WITH CELLULAR FUSION
PROTEIN AMINO ACID GLYCOSYLATION
GPI ANCHOR METABOLIC PROCESS
ATTACHMENT OF GPI ANCHOR TO PROTEIN
PHOSPHOINOSITIDE METABOLIC PROCESS
RHO PROTEIN SIGNAL TRANSDUCTION
REGULATION OF RAS PROTEIN SIGNAL TRANSDUCTION
REGULATION OF CELL SIZE
REGULATION OF SMALL GTPASE MEDIATED SIGNAL TRANSDUCTION
NEGATIVE REGULATION OF RAS PROTEIN SIGNAL TRANSDUCTION
90S PRERIBOSOME
CYTOKINESIS, COMPLETION OF SEPARATION
CYTOKINESIS
CYTOKINETIC CELL SEPARATION
REGULATION OF TRANSLATION
DI-, TRI-VALENT INORGANIC CATION TRANSPORT
PHOSPHOLIPID TRANSPORT
CELL WALL CHITIN METABOLIC PROCESS
SYNAPSIS
COFACTOR TRANSPORT

Figure S7

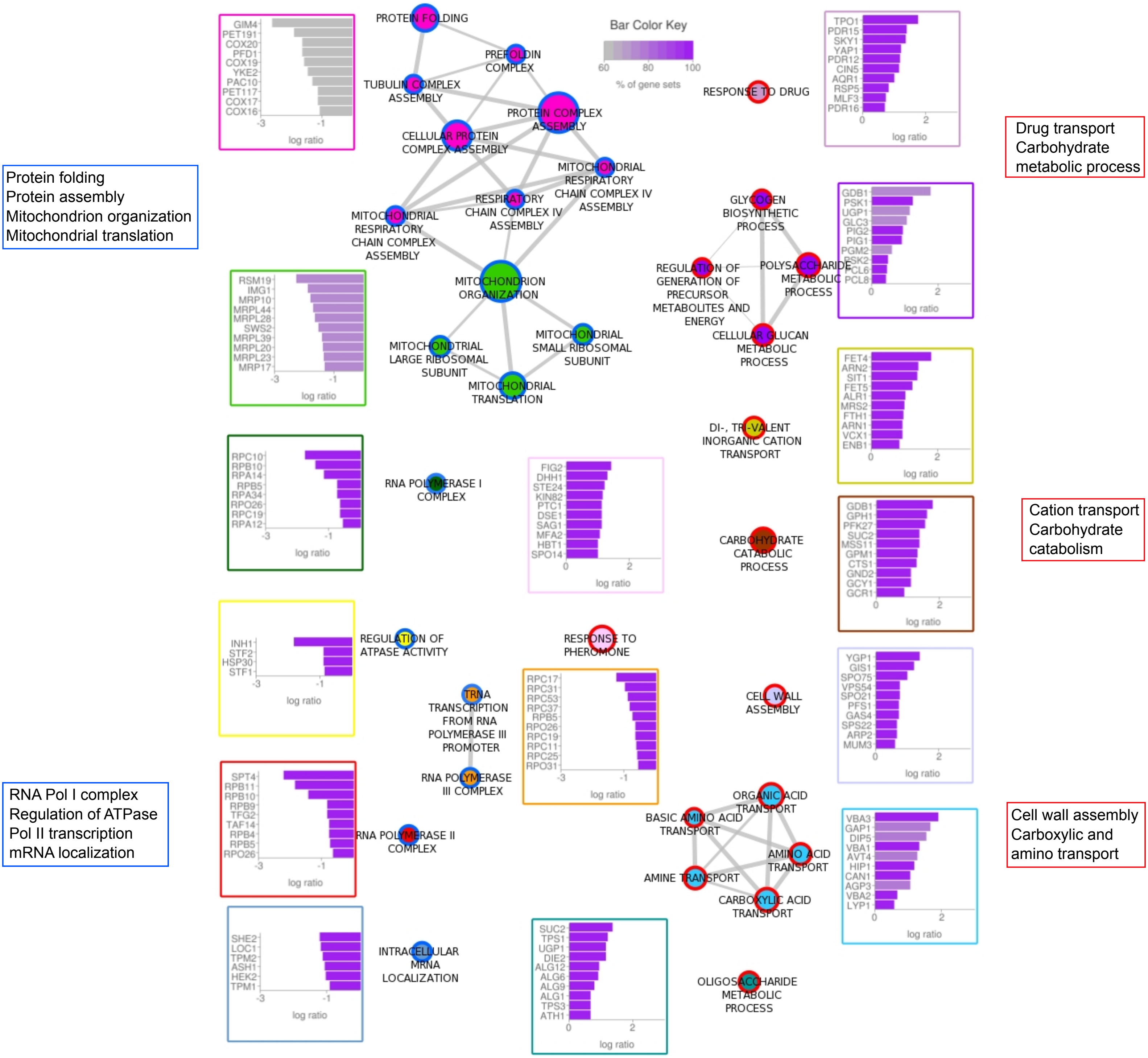

Protein folding
Protein assembly
Mitochondrion organization
Mitochondrial translation
RNA Pol I complex
Regulation of ATPase
Pol II transcription
mRNA localization
Drug transport
Carbohydrate metabolic process
Cation transport
Carbohydrate catabolism
Cell wall assembly
Carboxylic and amino transport
Bar Color Key
60 80 100
% of gene sets
PROTEIN FOLDING
PREFOLDIN COMPLEX
TUBULIN COMPLEX ASSEMBLY
PROTEIN COMPLEX ASSEMBLY
CELLULAR PROTEIN COMPLEX ASSEMBLY
MITOCHONDRIAL RESPIRATORY CHAIN COMPLEX IV ASSEMBLY
RESPIRATORY CHAIN COMPLEX IV ASSEMBLY
MITOCHONDRIAL RESPIRATORY CHAIN COMPLEX ASSEMBLY
MITOCHONDRION ORGANIZATION
MITOCHONDRIAL LARGE RIBOSOMAL SUBUNIT
MITOCHONDRIAL SMALL RIBOSOMAL SUBUNIT
MITOCHONDRIAL TRANSLATION
RNA POLYMERASE I COMPLEX
REGULATION OF ATPASE ACTIVITY
TRNA TRANSCRIPTION FROM RNA POLYMERASE III PROMOTER
RNA POLYMERASE III COMPLEX
RNA POLYMERASE II COMPLEX
INTRACELLULAR MRNA LOCALIZATION
RESPONSE TO PHEROMONE
RESPONSE TO DRUG
GLYCOGEN BIOSYNTHETIC PROCESS
REGULATION OF GENERATION OF PRECURSOR METABOLITES AND ENERGY
POLYSACCHARIDE METABOLIC PROCESS
CELLULAR GLUCAN METABOLIC PROCESS
DI-, TRI-VALENT INORGANIC CATION TRANSPORT
CARBOHYDRATE CATABOLIC PROCESS
CELL WALL ASSEMBLY
ORGANIC ACID TRANSPORT
BASIC AMINO ACID TRANSPORT
AMINO ACID TRANSPORT
AMINE TRANSPORT
CARBOXYLIC ACID TRANSPORT
OLIGOSACCHARIDE METABOLIC PROCESS
GIM4 PET191 COX20 PFD1 COX19 YKE2 PAC10 PET117 COX17 COX16
-3 -1 log ratio
RSM19 IMG1 MRP10 MRPL44 MRPL28 SWS2 MRPL39 MRPL20 MRPL23 MRP17
-3 -1 log ratio
RPC10 RPB10 RPA14 RPB5 RPA34 RPO26 RPC19 RPA12
-3 -1 log ratio
INH1 STF2 HSP30 STF1
-3 -1 log ratio
SPT4 RPB11 RPB10 RPB9 TFG2 TAF14 RPB4 RPB5 RPO26
-3 -1 log ratio
SHE2 LOC1 TPM2 ASH1 HEK2 TPM1
-3 -1 log ratio
FIG2 DHH1 STE24 KIN82 PTC1 DSE1 SAG1 MFA2 HBT1 SPO14
0 2 log ratio
RPC17 RPC31 RPC53 RPC37 RPB5 RPO26 RPC19 RPC11 RPC25 RPO31
-3 -1 log ratio
SUC2 TPS1 UGP1 DIE2 ALG12 ALG6 ALG9 ALG1 TPS3 ATH1
0 2 log ratio
TPO1 PDR15 SKY1 YAP1 PDR12 CIN5 AQR1 RSP5 MLF3 PDR16
0 2 log ratio
GDB1 PSK1 UGP1 GLC3 PIG2 PIG1 PGM2 PSK2 PCL6 PCL8
0 2 log ratio
FET4 ARN2 SIT1 FET5 ALR1 MRS2 FTH1 ARN1 VCX1 ENB1
0 2 log ratio
GDB1 GPH1 PFK27 SUC2 MSS11 GPM1 CTS1 GND2 GCY1 GCR1
0 2 log ratio
YGP1 GIS1 SPO75 VPS54 SPO21 PFS1 GAS4 SPS22 ARP2 MUM3
0 2 log ratio
VBA3 GAP1 DIP5 VBA1 AVT4 HIP1 CAN1 AGP3 VBA2 LYP1
0 2 log ratio

Figure S8

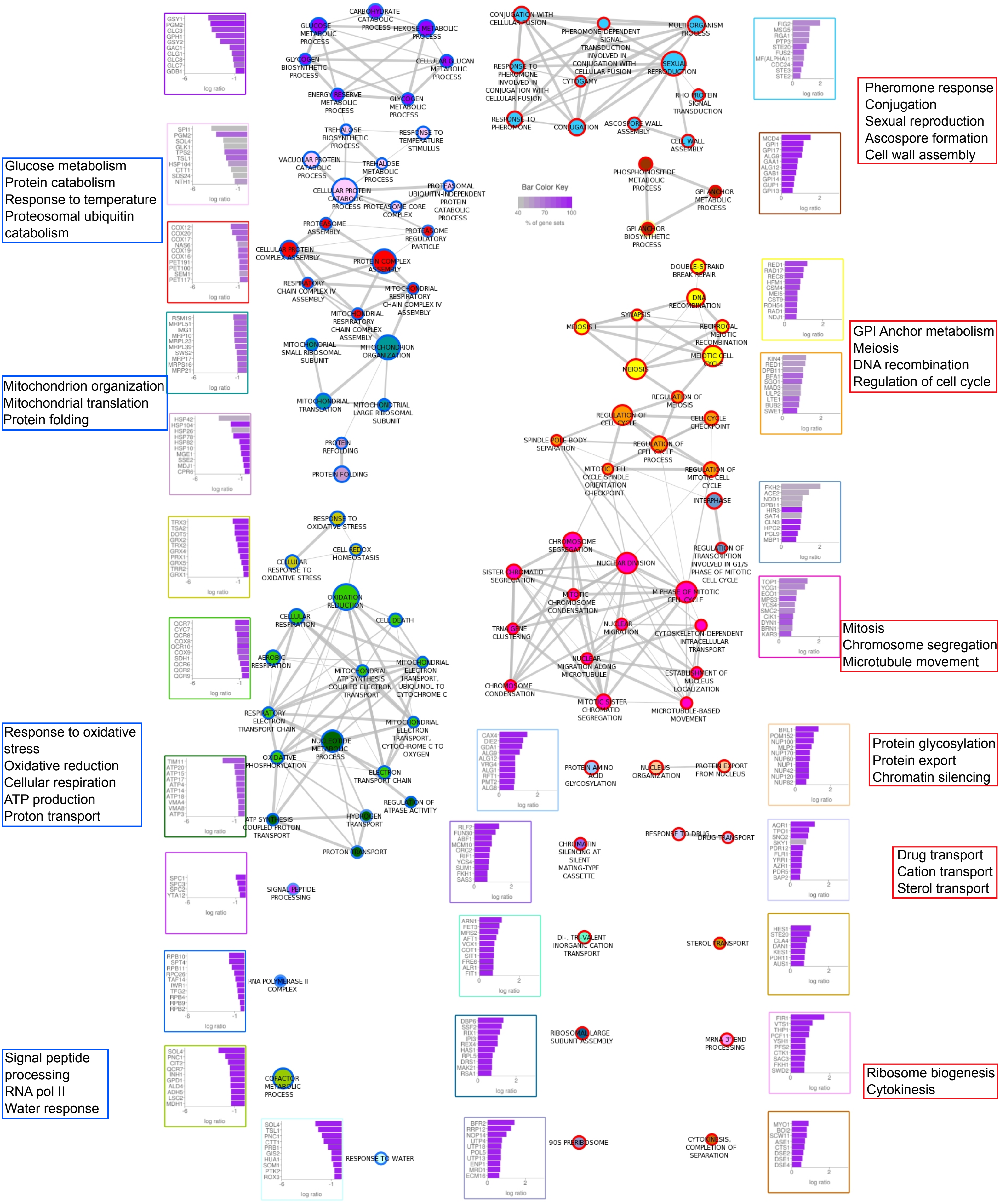

Glucose metabolism
Protein catabolism
Response to temperature
Proteosomal ubiquitin catabolism
Mitochondrion organization
Mitochondrial translation
Protein folding
Response to oxidative stress
Oxidative reduction
Cellular respiration
ATP production
Proton transport
Signal peptide processing
RNA pol II
Water response
Pheromone response
Conjugation
Sexual reproduction
Ascospore formation
Cell wall assembly
GPI Anchor metabolism
Meiosis
DNA recombination
Regulation of cell cycle
Mitosis
Chromosome segregation
Microtubule movement
Protein glycosylation
Protein export
Chromatin silencing
Drug transport
Cation transport
Sterol transport
Ribosome biogenesis
Cytokinesis
Bar Color Key
40
70
100
% of gene sets
log ratio
CARBOHYDRATE CATABOLIC PROCESS
GLUCOSE METABOLIC PROCESS
HEXOSE METABOLIC PROCESS
GLYCOGEN BIOSYNTHETIC PROCESS
CELLULAR GLUCAN METABOLIC PROCESS
ENERGY RESERVE METABOLIC PROCESS
GLYCOGEN METABOLIC PROCESS
TREHALOSE BIOSYNTHETIC PROCESS
RESPONSE TO TEMPERATURE STIMULUS
VACUOLAR PROTEIN CATABOLIC PROCESS
TREHALOSE METABOLIC PROCESS
CELLULAR PROTEIN CATABOLIC PROCESS
PROTEASOMAL UBIQUITIN-INDEPENDENT PROTEIN CATABOLIC PROCESS
PROTEASOME CORE COMPLEX
PROTEASOME ASSEMBLY
PROTEASOME REGULATORY PARTICLE
CELLULAR PROTEIN COMPLEX ASSEMBLY
PROTEIN COMPLEX ASSEMBLY
RESPIRATORY CHAIN COMPLEX IV ASSEMBLY
MITOCHONDRIAL RESPIRATORY CHAIN COMPLEX IV ASSEMBLY
MITOCHONDRIAL RESPIRATORY CHAIN COMPLEX ASSEMBLY
MITOCHONDRIAL SMALL RIBOSOMAL SUBUNIT
MITOCHONDRION ORGANIZATION
MITOCHONDRIAL TRANSLATION
MITOCHONDRIAL LARGE RIBOSOMAL SUBUNIT
PROTEIN REFOLDING
PROTEIN FOLDING
RESPONSE TO OXIDATIVE STRESS
CELL REDOX HOMEOSTASIS
CELLULAR RESPONSE TO OXIDATIVE STRESS
OXIDATION REDUCTION
CELLULAR RESPIRATION
CELL DEATH
AEROBIC RESPIRATION
MITOCHONDRIAL ATP SYNTHESIS COUPLED ELECTRON TRANSPORT
MITOCHONDRIAL ELECTRON TRANSPORT, UBIQUINOL TO CYTOCHROME C
RESPIRATORY ELECTRON TRANSPORT CHAIN
NUCLEOTIDE METABOLIC PROCESS
MITOCHONDRIAL ELECTRON TRANSPORT, CYTOCHROME C TO OXYGEN
OXIDATIVE PHOSPHORYLATION
ELECTRON TRANSPORT CHAIN
REGULATION OF ATPASE ACTIVITY
ATP SYNTHESIS COUPLED PROTON TRANSPORT
HYDROGEN TRANSPORT
PROTON TRANSPORT
SIGNAL PEPTIDE PROCESSING
RNA POLYMERASE II COMPLEX
COFACTOR METABOLIC PROCESS
RESPONSE TO WATER
CONJUGATION WITH CELLULAR FUSION
PHEROMONE-DEPENDENT SIGNAL TRANSDUCTION INVOLVED IN CONJUGATION WITH CELLULAR FUSION
MULTI ORGANISM PROCESS
SEXUAL REPRODUCTION
RESPONSE TO PHEROMONE INVOLVED IN CONJUGATION WITH CELLULAR FUSION
CYTOGAMY
RHO PROTEIN SIGNAL TRANSDUCTION
RESPONSE TO PHEROMONE
CONJUGATION
ASCOSPORE WALL ASSEMBLY
CELL WALL ASSEMBLY
PHOSPHOINOSITIDE METABOLIC PROCESS
GPI ANCHOR METABOLIC PROCESS
GPI ANCHOR BIOSYNTHETIC PROCESS
DOUBLE-STRAND BREAK REPAIR
DNA RECOMBINATION
SYNAPSIS
MEIOSIS I
RECIPROCAL MEIOTIC RECOMBINATION
MEIOTIC CELL CYCLE
MEIOSIS
REGULATION OF MEIOSIS
REGULATION OF CELL CYCLE
CELL CYCLE CHECKPOINT
SPINDLE POLE BODY SEPARATION
REGULATION OF CELL CYCLE PROCESS
MITOTIC CELL CYCLE SPINDLE ORIENTATION CHECKPOINT
REGULATION OF MITOTIC CELL CYCLE
INTERPHASE
CHROMOSOME SEGREGATION
SISTER CHROMATID SEGREGATION
NUCLEAR DIVISION
REGULATION OF TRANSCRIPTION INVOLVED IN G1/S PHASE OF MITOTIC CELL CYCLE
MITOTIC CHROMOSOME CONDENSATION
M PHASE OF MITOTIC CELL CYCLE
TRNA GENE CLUSTERING
NUCLEAR MIGRATION
CYTOSKELETON-DEPENDENT INTRACELLULAR TRANSPORT
NUCLEAR MIGRATION ALONG MICROTUBULE
ESTABLISHMENT OF NUCLEUS LOCALIZATION
CHROMOSOME CONDENSATION
MITOTIC SISTER CHROMATID SEGREGATION
MICROTUBULE-BASED MOVEMENT
PROTEIN AMINO ACID GLYCOSYLATION
NUCLEUS ORGANIZATION
PROTEIN EXPORT FROM NUCLEUS
CHROMATIN SILENCING AT SILENT MATING-TYPE CASSETTE
RESPONSE TO DRUG
DRUG TRANSPORT
DI-, TRI-VALENT INORGANIC CATION TRANSPORT
STEROL TRANSPORT
RIBOSOMAL LARGE SUBUNIT ASSEMBLY
MRNA 3'-END PROCESSING
90S PRERIBOSOME
CYTOKINESIS, COMPLETION OF SEPARATION

Figure S9

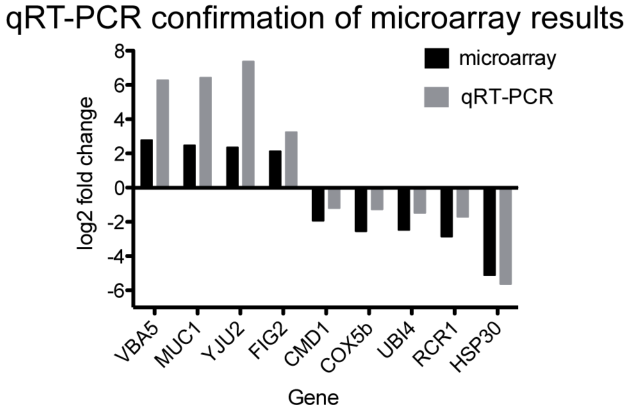
qRT-PCR confirmation of microarray results
microarray
qRT-PCR
log2 fold change
8
6
4
2
0
-2
-4
-6
VBA5
MUC1
YJU2
FIG2
CMD1
COX5b
UBI4
RCR1
HSP30
Gene

Figure S10

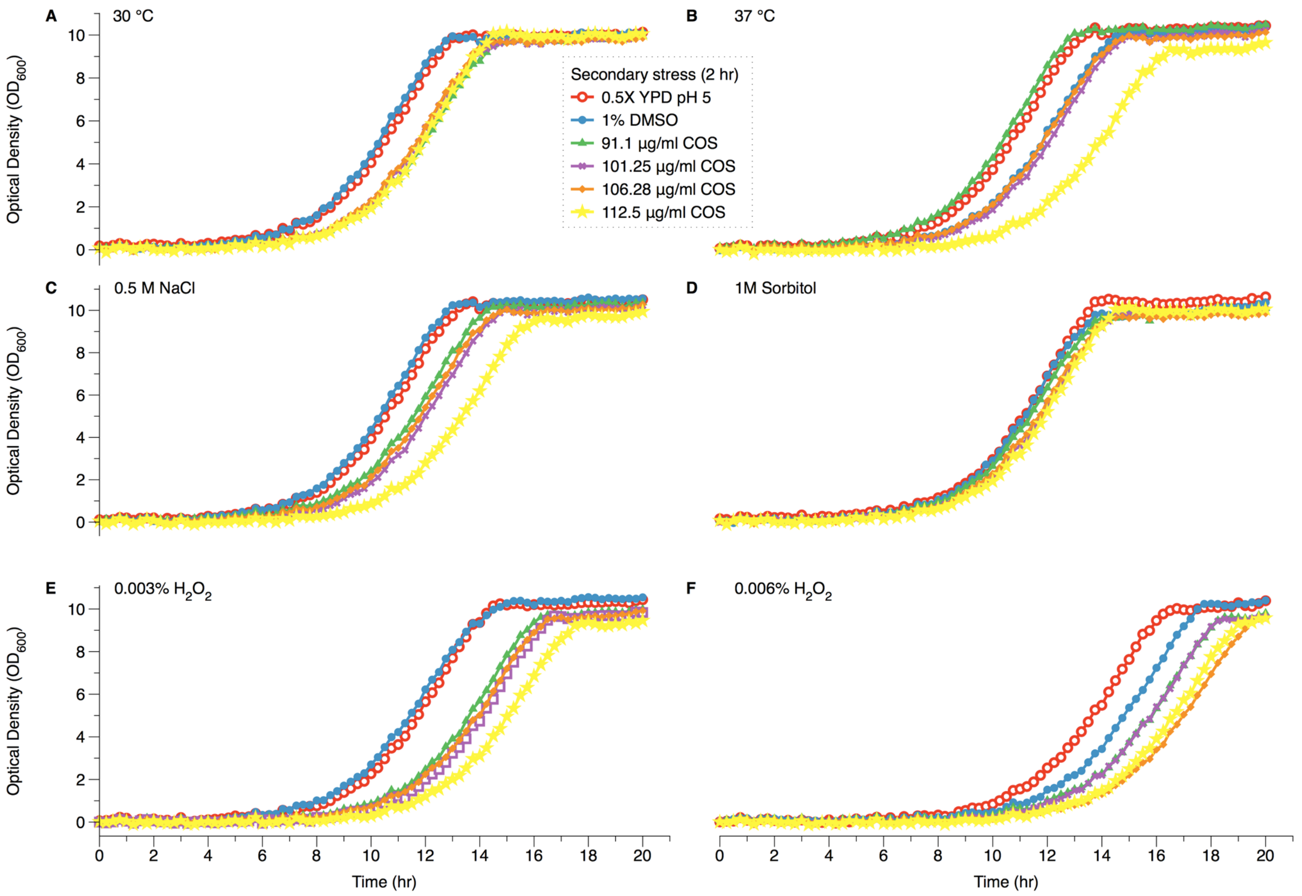
A
30 °C
B
37 °C
C
0.5 M NaCl
D
1M Sorbitol
E
0.003% H2O2
F
0.006% H2O2
Optical Density (OD600)
Time (hr)
Secondary stress (2 hr)
0.5X YPD pH 5
1% DMSO
91.1 µg/ml COS
101.25 µg/ml COS
106.28 µg/ml COS
112.5 µg/ml COS

Figure S11
